# Decoding chromosomal instability insights in CRC by integrating omics and patient-derived organoids

**DOI:** 10.1186/s13046-025-03308-8

**Published:** 2025-02-28

**Authors:** Federica Papaccio, Manuel Cabeza-Segura, Blanca García-Micó, Francisco Gimeno-Valiente, Sheila Zúñiga-Trejos, Valentina Gambardella, María Fernanda Gutiérrez‐Bravo, Carolina Martinez‐Ciarpaglini, Pilar Rentero‐Garrido, Tania Fleitas, Susana Roselló, Juan Antonio Carbonell-Asins, Marisol Huerta, David Moro-Valdezate, Desamparados Roda, Noelia Tarazona, Manuel M. Sánchez del Pino, Andrés Cervantes, Josefa Castillo

**Affiliations:** 1 Department of Medicine, Surgery and Dentistry “Scuola Medica Salernitana”, University of Salerno, Via S. Allende, 84081 Baronissi, Italy; 2 Department of Medical Oncology, Hospital Clínico Universitario de Valencia, INCLIVA Biomedical Research Institute, University of Valencia, Avda. Blasco Ibañez 17, 46010 Valencia, Spain; 3 Centro de Investigación Biomédica en Red (CIBERONC), Instituto de Salud Carlos III, 28029 Madrid, Spain; 4 Cancer Evolution and Genome Instability Laboratory, University College London Cancer Institute, London, UK; 5 Bioinformatic Unit, INCLIVA Biomedical Research Institute, Avda. Menéndez y Pelayo 3, 46010 Valencia, Spain; 6 Experimental and Applied Biomedicine Research Group, Health Sciences Faculty, Universidad Particular Internacional SEK (UISEK), Quito, 170302 Ecuador; 7 Department of Pathology, Hospital Clínico Universitario, INCLIVA Biomedical Research Institute, University of Valencia, Avda. Blasco Ibañez 17, 46010 Valencia, Spain; 8 Precision Medicine Unit, INCLIVA Biomedical Research Institute, Avda. Menéndez y Pelayo 4, 46010 Valencia, Spain; 9 Biostatistic Unit, INCLIVA Biomedical Research Institute, Avda. Menéndez y Pelayo 3, 46010 Valencia, Spain; 10 Department of General Surgery, INCLIVA Biomedical Research Institute, Hospital Clínico Universitario de Valencia, University of Valencia, Valencia, Spain; 11 Institute of Biotechnology and Biomedicine (BIOTECMED), University of Valencia, 46100 Burjassot, Spain; 12 Department of Biochemistry and Molecular Biology, University of Valencia, 46100 Burjassot, Spain

**Keywords:** Chromosomal instability, Colorectal cancer, Multi-omics, Mass spectrometry-based proteomics, Patient-derived organoids

## Abstract

**Background:**

Chromosomal instability (CIN) is involved in about 70% of colorectal cancers (CRCs) and is associated with poor prognosis and drug resistance. From a clinical perspective, a better knowledge of these tumour’s biology will help to guide therapeutic strategies more effectively.

**Methods:**

We used high-density chromosomal microarray analysis to evaluate CIN level of patient-derived organoids (PDOs) and their original mCRC tissues. We integrated the RNA-seq and mass spectrometry-based proteomics data from PDOs in a functional interaction network to identify the significantly dysregulated processes in CIN. This was followed by a proteome-wGII Pearson correlation analysis and an in silico validation of main findings using functional genomic databases and patient-tissues datasets to prioritize the high-confidence CIN features.

**Results:**

By applying the weighted Genome Instability Index (wGII) to identify CIN, we classified PDOs and demonstrated a good correlation with tissues. Multi-omics analysis showed that our organoids recapitulated genomic, transcriptomic and proteomic CIN features of independent tissues cohorts. Thanks to proteotranscriptomics, we uncovered significant associations between mitochondrial metabolism and epithelial-mesenchymal transition in CIN CRC PDOs. Correlating PDOs wGII with protein abundance, we identified a subset of proteins significantly correlated with CIN. Co-localisation analysis in PDOs strengthened the putative role of IPO7 and YAP, and, through in silico analysis, we found that some of the targets give significant dependencies in cell lines with CIN compatible status.

**Conclusions:**

We first demonstrated that PDO models are a faithful reflection of CIN tissues at the genetic and phenotypic level. Our new findings prioritize a subset of genes and molecular processes putatively required to cope with the burden on cellular fitness imposed by CIN and associated with disease aggressiveness.

**Supplementary Information:**

The online version contains supplementary material available at 10.1186/s13046-025-03308-8.

## Introduction

Chromosomal instability (CIN), defined by the ongoing rate of chromosome missegregation, is a recognized hallmark of cancer, conferring the necessary phenotypic plasticity for cells to survive in stressful conditions as well as increasing heterogeneity, promoting tumour evolution [1]. 70% of colorectal cancers (CRCs) display CIN [2], associated with poor prognosis [3]. However, strategies specifically targeting this tumour type are lacking, and patients are neither treated nor stratified based on this feature.

CIN can be triggered by mutations or treatments that impair the cellular processes involved in accurate chromosome segregation; telomere alterations and DNA-repair damage can also contribute to inducing CIN. In addition, chromosomal breaks and rearrangements induced by CIN and chromothripsis can help alter genome integrity and increase CIN [4–7]. These complex alterations constitute a significant burden for cancer cells, as normal cells fail to survive even small alterations [8]. Somewhat paradoxically, accumulating additional alterations can improve tumourigenesis, possibly due to emerging mechanisms that help the cell to overcome fitness stress [9], while an excess of CIN can in turn increase cell stress until a point of no return thereby inducing cell death [10, 11]. Therefore, the knowledge of the mechanisms that help to cope with CIN stress could lead to develop innovative treatment strategies, and omics technologies, able to explore the impact of chromosomal abnormalities on a large scale, can certainly play a key role in this endeavour.

The dynamic nature of CIN requires the use of proper functional models. Indeed, although 2D cancer cell lines have been extensively used to study the dynamics of CIN in cancer, they tend to accumulate genome abnormalities per se and do not represent intratumoural heterogeneity. Tumour patient-derived organoids (PDOs) currently provide the most faithful depiction of human cancer and could represent the tool to fill the CIN knowledge gap. A few studies employed organoids as CIN models. Indeed, CRC PDOs were shown to widely display CIN, and that despite mitotic errors led to cell death, some PDOs were largely insensitive to them [12], while radioresistant rectal organoids displayed less CIN than sensitive ones [13]. Ovarian cancer PDOs were shown to be good models of CIN in terms of genomics and transcriptomics [14], while oesophageal cancer PDOs were employed to investigate the causes of CIN [15]. Investing the poor defined molecular mechanism that underlie CIN in PDOs is crucial to increase our knowledge of this complex phenomenon, as until recently all we know came from 2D cell lines or tissues. However, more data are needed to confirm that the CIN profile could be truly recapitulated by organoids, at both genotype and phenotype level and none of the studies conducted so far with PDOs focused on the processes that are ongoing to help endure CIN.

We previously demonstrated that PDOs from patients with advanced CRC faithfully recapitulated the genome and transcriptome of tissues, and through an integrated proteotranscriptomic approach we could identify promising biomarkers of response/resistance to both standard and non-standard drugs [16]. Here, we used the weighted Genome Instability Index (wGII) to classify our PDO models according to CIN [17]. We demonstrated that they reproduce the CIN phenotype of tissues in terms of genome, transcriptome and proteome. Proteotranscriptomics uncovered a significant relationship between metabolic rewiring and epithelial-mesenchymal transition (EMT) in CIN + CRC PDOs. Moreover, a proteome-wGII correlation reinforced these processes pointing more specifically to enhanced mitochondrial metabolism, with a significant increase in activities related to acyl-CoA species, and to the activation of YAP signalling in CIN. Using omics and functional genomic databases, we prioritised a subset of these proteins and molecular processes putatively relevant in CIN. Taken together, our results add knowledge of the molecular mechanisms that could be operating in high-CIN CRC PDOs, helping them withstand the stressful conditions imposed by this phenomenon, and which constitute putative therapeutic targets.

## Methods

Detailed methods are described in Supplementary Methods. All reagents and tools are listed in Table S1.

### Ethics

The study was approved by the Hospital Clínico Universitario de Valencia Ethics Committee (2018/063, 2021/083) in compliance with the Declaration of Helsinki. All patients provided written informed consent.

### Tissue processing and organoid culture

Fresh tissues were processed as previously published [16]. Supplementary table S2 shows the main features and analysis performed for each of the organoids employed in this paper.

### Copy number and CIN status determination

Cytoscan HD was performed on PDOs and tissues according to the manufacturer protocol. Data were analysed with ChAS and IGV (v. 3.0).

Weighted Genome Instability Index (wGII) was calculated as published elsewhere [17]. Genomic co-ordinates of gain/lost regions retrieved from ChAS were used to estimate the total of copy number alterations (CNA) as base pair (bp), normalized for chromosome length.

### RNA-Sequencing (RNA-seq) and Proteomics analysis by LC–MS/MS-SWATH

RNA-seq and quantitative proteomics were performed as previously published [16]. See Supplementary Methods for details.

Differential gene expression analysis was conducted with the DESeq2 v1.34 package with RStudio. GSEA was used for hallmarks analysis. Unsupervised hierarchical cluster analysis based on the “EMT” gene set from the Molecular Signatures Database (MSigDB, GSEA) was done using a Euclidean distance measure and Ward linkage. Motif analysis has been conducted using FASTQ files to run ISMARA software (https://ismara.unibas.ch/mara/). Obtained motif z-values have been filtered out excluding those lower than 1.5.

Differential expression analysis based on SWATH normalized protein areas was performed. Functional analysis was conducted using STRING database and Cytoscape StringApp. The correlation between CIN and protein abundance was analysed by calculating the Pearson coefficient. To identify mitochondrial proteins, we used information from UniProt (subcellular location and GO Cellular component). Proteins containing the term ‘Mitochondria’ in either of these two entries were considered to be mitochondrial proteins, although they may also be in other locations. Differential proteins according to RNAseq or proteomic (differential and Pearson correlation analysis) data are considered.

### Immunofluorescence staining of PDOs paraffin sections

PDOs domes were collected in 4% neutral buffered formalin and paraffin embedded as previously described [16], 4 um slides were cut, dewaxed and sodium citrate antigen retrieval was performed (Target Retrieval Solution, Citrate pH 9, S236784-2) followed by blocking (Dako-REAL™, Dako, cat. No. S2023). PDO’ slides were incubated with the following primary antibodies in EnVision FLEX antibody diluent (Dako, cat. No. K8006): ActYAP1 (abcam; ab205270; 1:200), IPO7 (Santa Cruz; sc-365231; 1:50), acetylated-lysine (Cell signalling; #9441; 1:200). After washing three times with PBS samples were incubated with corresponding Alexa 488- and Alexa 647-conjugated secondary antibodies (Invitrogen; A11001, A31571; 1:500) and mounted in Prolong Gold Antifade Mountant with DNA Stain DAPI (Invitrogen; P36941). Samples were imaged on a confocal microscope LEICA TCS-SP8. Representative images were acquired and shown as Z‐projections, single slices or XZ cross sections. Image analysis has been performed with CellProfiler and ImageJ software.

### Statistical analyses

The significance threshold of all statistical analyses was set at *p*-value below 0.05. wGII correlation between PDOs and matched fresh tissue was conducted with linear regression analysis. The PDOs-TCGA transcriptomic comparison was performed with Chi-square test. The correlation between CIN and protein abundance was analysed by calculating the Pearson coefficient. We used the Pearson function of Microsoft Excel spreadsheet with the median normalized and log2 transformed data for each protein and the wGII value for each sample as function inputs. Venn diagrams were used to visualize differences between categorical groups. Statistical analyses related to publicly datasets are described in the corresponding section.

### Publicly available datasets analysis

For cell lines dependencies, CIN + and CIN- cell lines were analysed for combined CRISPR/Cas9 DepMap Public 23Q4 + Score and Chronos datasets and RNA-interference combined Achilles + DRIVE + Marcotte, DEMETER2 from DepMap portal (https://depmap.org/portal/), selecting genes from the Pearson signature proteins. The dependence score for each gene in CIN + vs CIN- was compared via multiple t-test. Data were represented with Volcano plots indicating the dependence score effect size. For drugs analysis the AUCs for each compound of the CTD^2 dataset from DepMap portal were compared for CIN + vs CIN- via multiple t-test.

Proteins from the Pearson signature were searched in the proteomic data from Zhang et al. [18] for CIN vs MSI annotated samples. Z-scores were calculated for each protein, and a clustering heatmap was built with pheatmap package. Fisher exact test was used to evaluate statistical significance of tissue categorization.

Kaplan–Meier plotter software (https://kmplot.com/analysis/) was used to evaluate the prognostic value of the prioritized targets. TNMplot web tool (https://tnmplot.com/analysis/) was used to compare gene expression in CRC tumour versus normal tissue using RNAseq data and normal tissues near the tumour area.

## Results

### PDOs reproduce CIN profile of tissues at genome and transcriptome level

CytoscanHD was conducted on PDOs and matched fresh tissues to detect CNA (Additional file S1). We included 9 patients/12 PDOs for which we have copy number data, a sample size similar to previous studies [12–15]. As we previously demonstrated with fewer models [16], they share the same copy number profile (Fig S1), and in most cases organoids were enriched in gains/losses compared to the tissues.

**Fig. 1:**
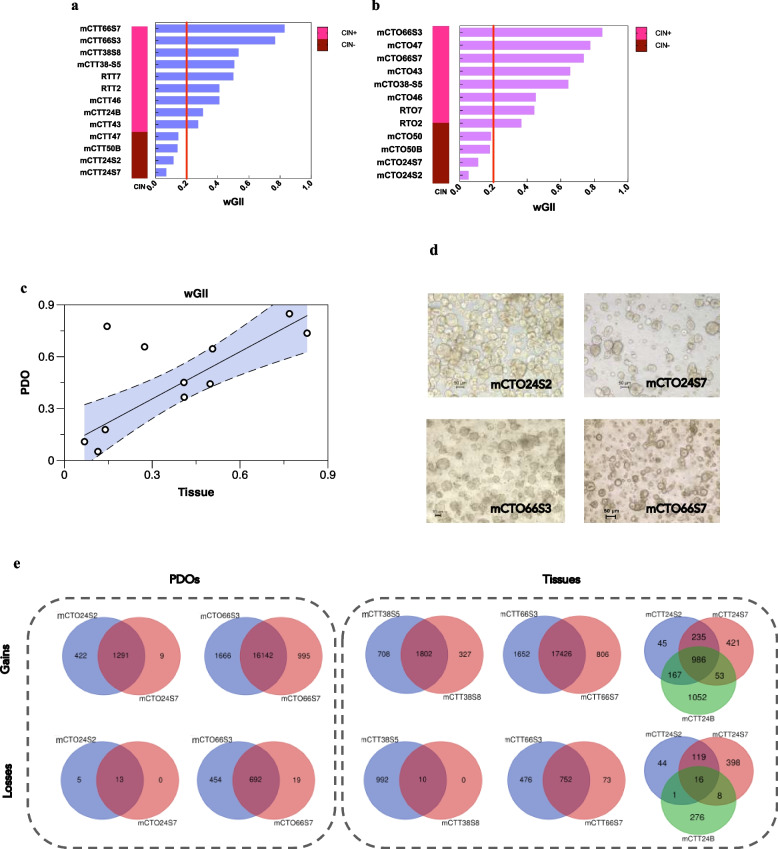
PDOs reproduce chromosomal instability (CIN) original tissue profile showing inter-metastasis heterogeneity. **a-b** wGII distribution in tissues (**a**) and PDOs (**b**) and consequent classification of samples in CIN + (wGII > 0·2) or CIN- (wGII < 0·2). **c** Correlation analysis between organoids and tissues wGII. The shaded area indicates the bounds of the 95% confidence limit of the regression after removing the outliers (> 2 standard deviations of the residuals). Fitting values *r* = 0·867, *p*-value = 0·001. **d** Representative bright-field microscopy images of PDOs lines generated from different metastasis of the same patient. Scale bar 50 μm. **e** Venn diagrams of gene-level copy number alterations in terms of gains and losses depicting inter-metastasis heterogeneity

The wGII was employed as a surrogate of CIN, as a wGII > 0·2 indicates the presence of CIN [17]. Across our cohort of twelve matched organoids and tissues (Table S2), 70% of samples displayed CIN (Fig. 1a-b), consistent with the literature, and PDO CIN had a positive correlation with tissue’ CIN (linear regression, r = 0·867, *p*-value = 0·001) (Fig. 1c). Nevertheless, two cases showed substantial discordance. For patient 43, the wGII was much higher in organoid than tissue, while in patient 47, tissue was negative while PDOs had one of the highest wGII, compatible with an extremely low tumour cells percentage [16].

Interestingly, CIN status can be evolutionarily acquired, as shown by patient 24, where organoids generated from two different synchronous liver metastases were CIN-, but the tissue obtained from a progressive brain metastasis was CIN + (Fig. 1a), indicating the acquisition of a more aggressive phenotype.

PDOs generated from different metastases of the same patient show morphological heterogeneity in culture (Fig. 1d). Using the comprehensive coverage of CytoscanHD which goes up to gene level, we assessed intra-patient copy number heterogeneity from different metastases, detecting significant differences in CNA (Fig. 1e). Unfortunately, this analysis was precluded for mCTO38S8 and for the brain metastasis of patient 24 where only tissue comparison was possible, due to lack of growth before obtaining sufficient DNA in the first case, and not at all for the second. We also detected mosaicism, confirming heterogeneity in terms of subclonal CNA (Fig. S2).

**Fig. 2:**
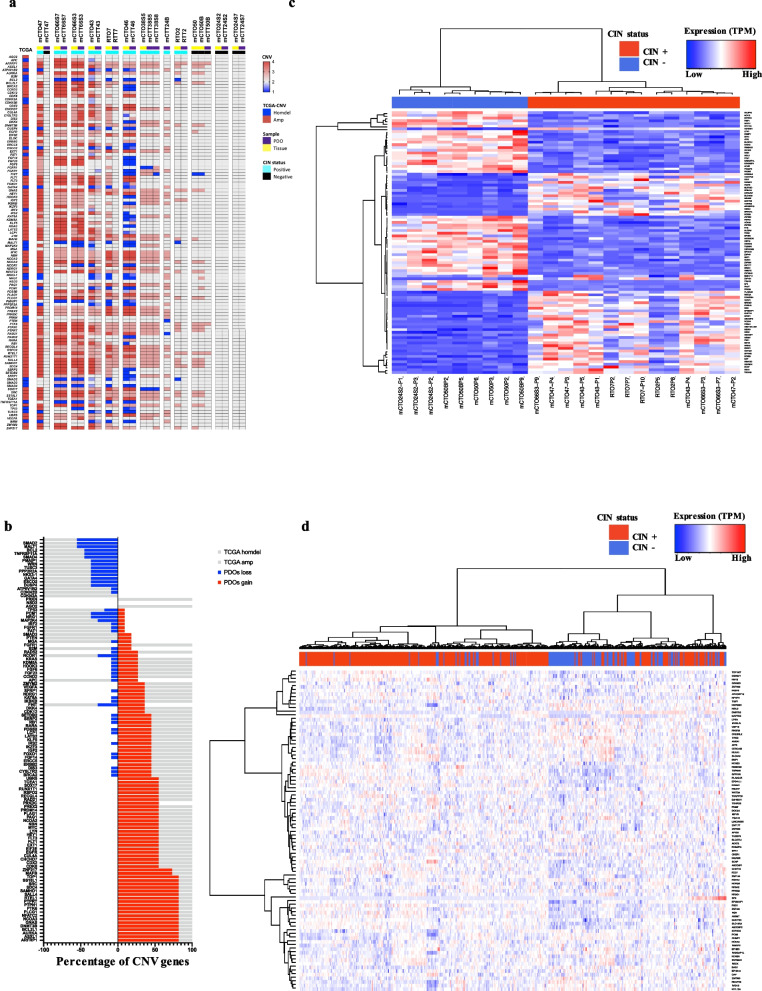
PDOs reflect the genomic and transcriptomic profile of CIN + TCGA tissues. **a** Heatmap representing CNV (in terms of copy number state where 2 = diploid; < 2 = loss; > 2 = gain) in frequently altered genes across TCGA. Each PDO line is classified as CIN + (light blue) or CIN- (black) and represented side by side with its corresponding tissue. **b** Frequency of gain/lost genes in PDOs cohort. Genes are selected from the TCGA. In grey cumulative frequencies of lost (negative %) or gained (positive %) genes in the TCGA. In blue/red cumulative frequencies of lost/gained genes in PDOs. **c** Hierarchical Clustering Heatmap showing the top 100 genes more differentially expressed between CIN + vs CIN- PDOs. The colour code indicates gene expression. Biological duplicates/triplicates of PDOs cultures shows a constant expression profile across different passages. The columns corresponding to each sample are indicated at the bottom while its corresponding CIN status is colour-coded above. **d** Top 100 differential expressed genes in our PDO’ cohort classify TCGA tissues (PanCancer COAD cohort) in terms of CIN. To the right-hand side of the figure, a scale indicates the colour code relative to the normalized TPM abundance. The colour code legend is the same as panel **a**

Next, lost/gained genes were matched with the most commonly reported within the TCGA. The gene list was extracted from CRC TCGA (CBioPortal) selecting genes altered in at least 1% of samples. These were compared with our PDOs-tissues cohort (Fig. 2a). The genes that most frequently present copy number gains/losses across the TCGA were found within our PDOs and confirmed in our tissues.

The most frequently gained/lost genes in our cohorts specifically are depicted in Fig. 2b. Among the amplified genes *AURKA* was gained in 80% of PDOs and has been associated with CIN as it regulates the function of centrosomes, spindles and kinetochores for mitotic progression [19], and *TOP1*, involved in the stabilization of long chromosomes [20]. Indeed, on histological slides from CIN and non-CIN subtypes, numerous mitotic figures were evident in both groups (between 6 to 18 per 5 high power fields (HPF) (Fig. S3a). However, atypical mitotic figures appeared to be more frequently observed in the CIN subtype (64% vs. 48%), supporting a phenotype indicative of mitotic spindle alterations in CIN PDOs. Among the lost genes were *PCM1* [21], involved in centrosome integrity maintenance, *TUSC3* [22], which can inhibit EMT, and *FHIT* [23], related with CIN. When we consider the pathogenic mutations of PDOs [16], mutant genes tend to have more copy number variations in CIN + organoids (Fig.S3b).

We previously showed that PDOs reproduced the transcriptomic profile of their original tissues [16] so here we hypothesized whether PDOs would reflect CIN + in an independent tissues’ cohort at the transcriptomic level. We computed differential expression analysis between all CIN + and CIN- PDOs. GSEA analysis showed that CIN + PDOs present an upregulation of MYC and E2F targets, “protein secretion” and “unfolded protein response” signatures as well as a downregulation of those related with “TNFalfa signaling”, “p53 pathway” and “apoptosis” signatures (Fig. S4). Subsequently, we found that using the 100 most differentially expressed genes (Fig. 2c; Additional file S2) we obtained a good classification of the COAD PanCancer cohort study in the TCGA (Fig. 2d; Chi-square test, *p* < 0·0001, Fig S5).

Many of the genes found in the top 100 were previously associated with CIN for their role in the dynamic of mitotic spindle (*NSMCE2*[24]), the kinetochore-microtubule complex (*FDG1*[25]) and the regulation of ploidy (*NUAK1* [26]). Another interesting gene was *NPHP4*, that encode a negative Hippo pathway regulator which has been associated with LATS1-induced chromosomal instability [27, 28].

Moreover, we contrast our data with previously described CIN signatures, and found that HET70 CIN signature independently classified our PDOs as CIN + or CIN- (pval < 0.001, Fisher’s exact test) (Fig. S6), aligning with the findings of Sheltzer [29].

### An integrative proteotranscriptomic approach to unravel differential processes in CIN+ and CIN- PDOs

**Fig. 3:**
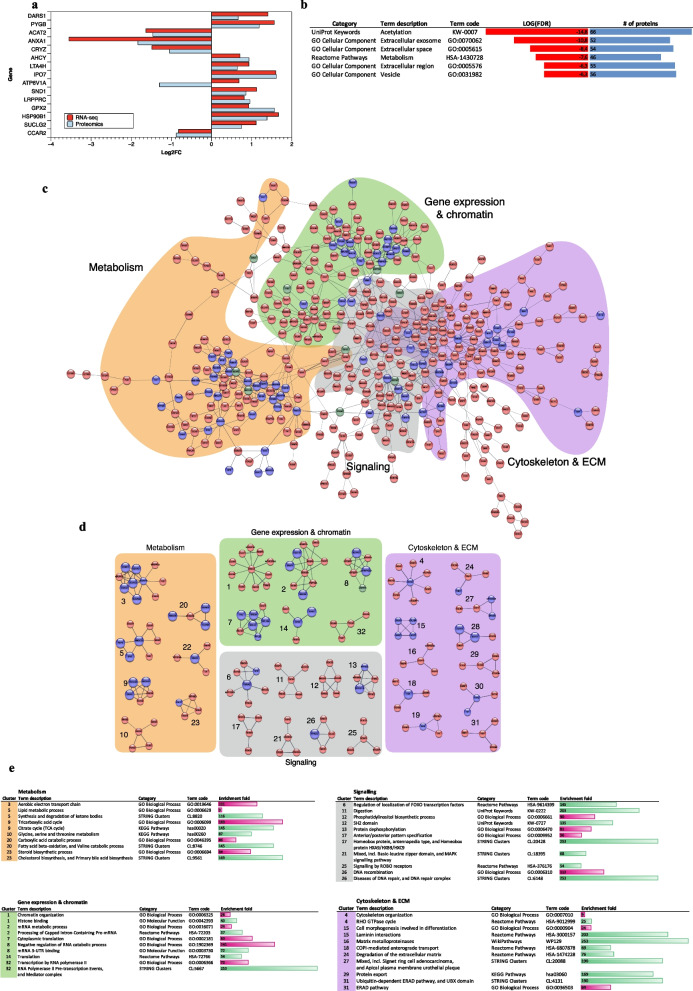
Integration of differential expression data at the proteomic and transcriptomic level between CIN + and CIN- PDOs. **a** Common genes differentially expressed at RNA and protein level between CIN + and CIN- PDOs. **b** Functional annotation of 101 genes detected as differentially expressed only at protein level. LogFDR and % of proteins enriched in the top 6 functional ontologies are represented. **c** Integrative functional network of proteins with significant differential expression in CIN positive vs CIN negative PDOs. RNA and protein data were fused in a single network where nodes in red correspond to proteins identified by RNA-seq only, in blue those identified by proteomics only and in green those identified by both omics. Four highly connected node modules were obtained, which have been shaded in different colours. Only networks with high confidence interactions (score 0·7 or higher) containing 4 or more nodes were considered. The name of each module tries to encompass the main functional terms in which they are enriched. **d** Clustered protein association network with the clusters grouped and shaded in the same colour code which they were assigned in the integration network analysis in panel **c**. Clustering was performed using the Markov clustering implementation in the Cytoscape-StringApp. In red nodes identified only by RNA, in blue nodes identified only by proteins, in green nodes identified by both RNA and proteins. Proteins included in the ‘acetylation’ term in panel **b** are marked with a bigger blue node. Clusters consisting of less than 4 nodes are omitted from the visualization. **e** Functional enrichment analysis of clusters within each module expressed as enrichment fold. GO Biological process are depicted in fuchsia, while other functional terms are depicted in green

Since PDOs were a good model of CIN + CRC at genomic and transcriptomic levels, we further investigated the molecular mechanisms underlying CIN in organoids, applying an integrated proteotranscriptomic strategy [16]. As we could perform SWATH-MS for a subset of PDOs, we computed differential gene expression analysis for the same models employed in proteomics (Table S2). We generated a proteomic dataset, identifying 116 proteins (with ≥ two peptides), 62 up- and 54 down-regulated in CIN + PDOs (Additional file S3), while in the transcriptomic dataset 1017 genes were differentially expressed, 644 up- and 373 downregulated (Additional file S2). Among them, 15 were commonly detected in both datasets (Fig. 3a) with highly correlated log2 FC values, while a modest correlation was observed for proteins showing significant differences only at RNA level (Fig. S7), highlighting the value of integrating both datasets in such studies. As many proteins (101/116) were differentially only at protein level, we performed a functional annotation study of these and found acetylation as the process containing the highest number of proteins [66/101] (Fig. 3b). These data align with the key role of post-translational modifications (PTMs) in gene expression regulation, with acetylation being particularly relevant in this dataset.

Our approach involved extracting common processes through an integrative network analysis using STRING database via Cytoscape [16]. We focused on the largest high confidence (score 0·7) functionally related network obtained (Fig. 3c). It contained 454 nodes, with 80 proteins, 364 RNAs and 10 differentially expressed genes detected at both protein and RNA level. Functional enrichment confirmed that most gene ontologies (GOs) were represented by both, with four highly connected modules annotated as ‘metabolism’, ‘cytoskeleton organization and extracellular matrix’, ‘gene expression and chromatin’ and ‘signaling’ (Fig. 3c). A topological clustering algorithm was applied to explore functional interactions between groups of nodes inside modules (Fig. 3d), with proteins included in the term ‘acetylation’ in panel b highlighted as larger blue nodes. Functional enrichment is shown in Fig. 3e, where the GO term and/or the first functional term with the lowest FDR has been selected for each cluster. The complete data are in Additional file S3. The ‘metabolic module’, containing seven clusters, showed a notable role for mitochondrial metabolism. Electron transport chain and mitochondrial ATP synthesis processes were enriched in cluster 3, including different subunits of complexes III and IV and the mitochondrial phosphate carrier SLC25A3, an essential component of the ATP synthasome [30]. Several enzymes involved in the tricarboxylic acid cycle (TCA) appeared in cluster 9, as SUCLA2 and SUCLG1, the two subunits of the Succinate-CoA ligase function in the TCA coupling the hydrolysis of succinyl-CoA to the synthesis of ATP. Strikingly, two other clusters (20 and 5) were functionally related to many processes involving acyl-CoA species. Cluster 20 was enriched in genes related to fatty acid beta-oxidation (FAO) and branched-chain amino acids (BCAA) degradation, while cluster 5 was associated with ketone body metabolism, which includes the Coenzyme A Synthase gene *COASY*. In the ‘cytoskeleton and extracellular matrix’ module nine clusters emerged. Cluster 4 contained a group of differential RNAs involved in actin cytoskeleton organization, integrated due to the upregulated hub protein CDC42, a small GTPase involved in the regulation of signalling pathways that control cell cycle progression, migration and morphology [31]. Cluster 15 was composed by a set of extracellular matrix glycoproteins including three laminins LAMB1, LAMC1 and LAMA1 together with COL18A1, all upregulated in CIN + . Cluster 27, meanwhile, included significantly reduced cell adhesion proteins well known for their involvement in gastrointestinal cancers such as EPCAM and CEACAM5. In the ‘gene expression and chromatin’ module mixed clusters 2, 7 and 8 were formed by RNA-binding proteins and ribosomal proteins involved in modulation of mRNA stability and translation of certain genes. Among these LRPPRC, a potential oncogene in multiple tumour types, detected at both RNA and protein level, which plays a role in mitochondria homeostasis [32]. We also detected upregulation of the two genes, *IGF2BP1/2*, that encode oncofoetal IGF2 mRNA-binding proteins, acting as RNA N^6^-methyladenosine modification readers. Recently, IGF2BP2 was shown to promote CRC progression by stabilizing oncogenic mRNAs, including YAP mRNA [33], a downstream nuclear effector of the Hippo signaling pathway with a role in the development and progression of multiple cancers. Finally, in ‘signaling’ module, cluster 6 showed the integration of genes and proteins related with the regulation of FOXO transcription factors localization, with a significant reduction in YWHAZ and SFN expression. These are members of 14–3-3 protein family, that associate with YAP leading to its translocation to the cytoplasm with consequent inhibition [34, 35]. Finally, cluster 13 contained protein phosphatases involved in chromosome segregation and spindle formation, and cluster 26 showed DNA-PK protein integrated with genes of the DNA repair machinery and related to chromosome organization.

### Proteomic analysis reveals laminin enrichment in CIN+ PDOs, consistent with an EMT transcriptomic profile

**Fig. 4:**
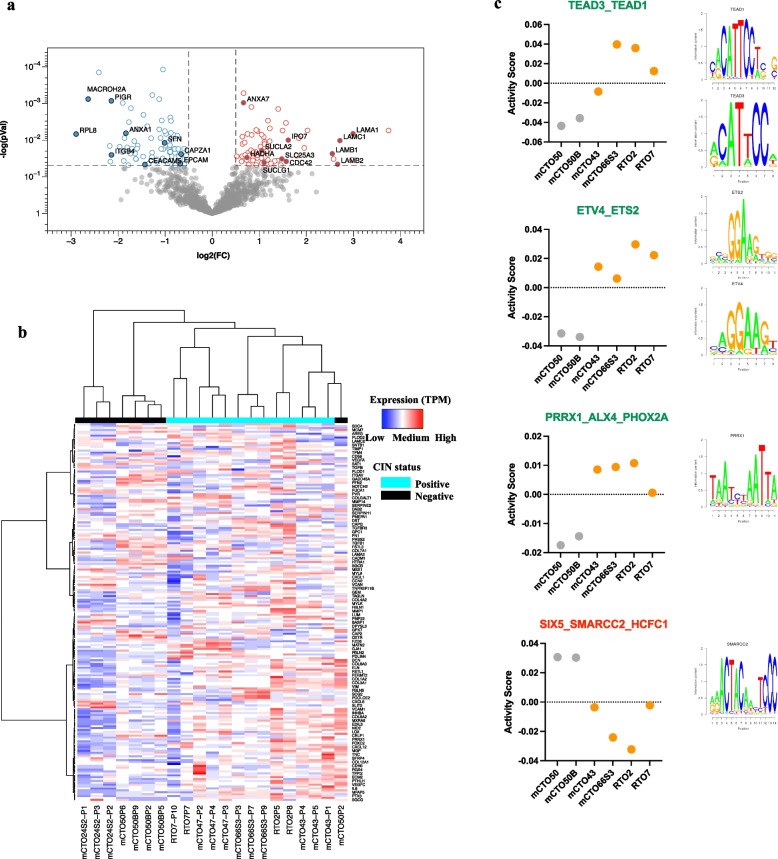
Association between CIN and EMT in PDOs at proteomic and transcriptomic level. **a** Volcano plot representation of differential protein expression in CIN + versus CIN- PDOs. Proteins with significantly increased or decreased expression (± 1·fivefold change, *p*-value < 0·05) are coloured red and blue, respectively. Some interesting proteins mentioned in the text are marked with solid dots and their names. **b** Unsupervised Hierarchical Clustering Heatmap representing the EMT expression signature in PDOs transcriptomic datasets. The colour code indicates gene expression. Biological duplicates/triplicates of PDOs cultures shows a constant expression profile across different passages. The columns corresponding to each sample and its corresponding CIN status are indicated at the bottom. To the right-hand side of the figure, a scale indicates the colour code relative to the normalized TPM abundance. (c) ISMARA-predicted transcription-factor-activity plot of the CIN- and CIN + models. *n* = 3 biological replicates

Among the clusters of the integrated analysis, cluster 15 drew our attention (Fig. 3d), as it includes a group of three laminins, LAMB1, LAMC1 and LAMA1, with the highest fold change in CIN (Fig. 4a). Laminins are key mediators of cell–cell and cell-basement membrane interaction and play a major role in cell adhesion, differentiation, and migration during embryogenesis [36, 37]. Several data highlight their role in EMT induction and in promoting cancer aggressiveness. Furthermore, CIN + PDOs displayed a significant reduction of EPCAM, in accordance with loss of epithelial phenotype. We also detected in CIN + PDOs enhanced expression of *CD44* gene, encoding a cell-surface receptor that plays a role in cell–cell interactions, cell adhesion and migration, which is increased in cancer cells with an EMT stem cell-like phenotype [38]. Intriguingly, some mitochondrial enzymes, which according to proteotranscriptomic analysis appeared enriched in CIN + PDOs, might themselves contribute to promote EMT as they are involved in the generation of metabolites (i.e. acetyl-CoA, α-ketoglutarate, succinyl-CoA) that serve as crucial ‘ink’ on epigenetic modifications [39, 40].

Collectively, these data suggested an association between CIN and EMT in our models. However, EMT is a complex, highly regulated and reversible process that requires the activation and silencing of many genes. Therefore, beyond specific changes, we analysed whether the phenotype of our models was globally compatible with an EMT phenotype. An unsupervised hierarchical clustering heatmap interrogating the expression of the MSigDB EMT signature, revealed that CIN + PDOs clustered together among those with the highest expression of EMT related genes (Fig. 4b). Futhermore, motif analysis via ISMARA software showed that CIN + organoids present a relevant higher activity of motives belonging to transcription factors involved in EMT, such as PRRX1, SMARCC2, ETV4 and ETS2, as well as TEAD3_TEAD1, member of the Hippo-YAP signaling pathway (Fig. 4c) as compared with CIN- organoids. In addition, ISMARA identified a higher activity in CIN + PDOs of CREB5_CREM_JUNB, CLOCK, NFKB1, NR4A3, GLIS3 which are related with mitochondrial function (fig. S8), as well as motives related with E2F transcription factors and others involved in genome instability (fig. S8).

### Proteome linked to CIN identify putative biomarkers and novel therapeutic targets

**Fig. 5:**
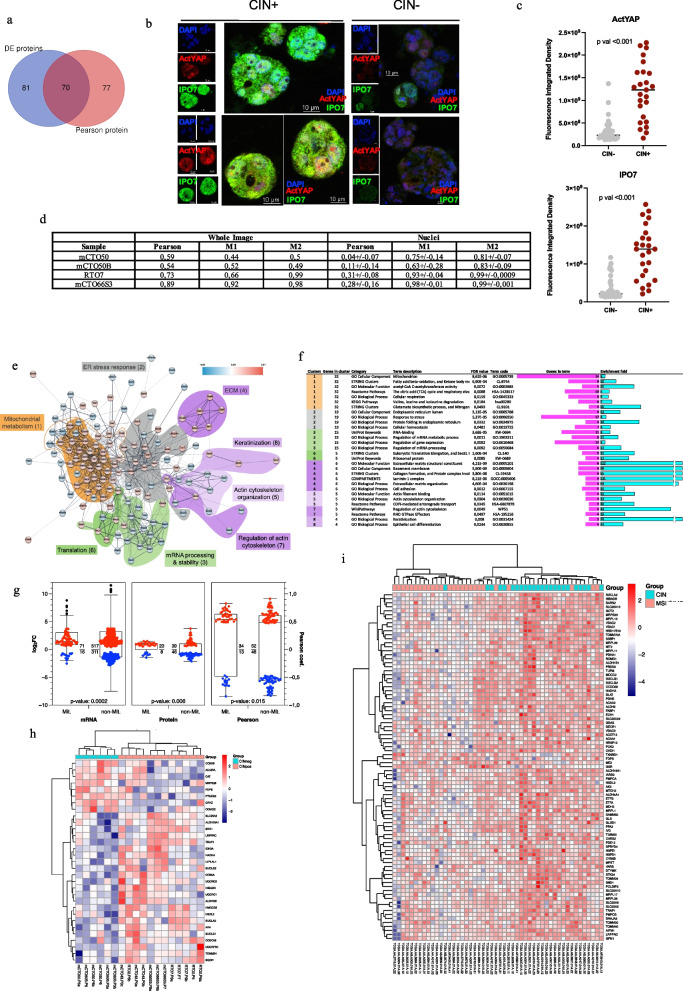
Functional analysis of proteins and processes that better correlate with CIN in PDOs. **a** Venn diagrams showing the number of proteins commonly detected via differential expression or Pearson analysis. **b** Confocal imaging of CIN + (left panel) and CIN- (right panel) organoids stained with DAPI (blue), anti-IPO7 (green) anti-activated YAP1 (non-phosphorylated, red). Representative images are shown. **c** Nuclear staining intensity for ActYAP and IPO7 single nuclei quantification by ImageJ. **d** Quantification of the degree of co-localization between ActYAP and IPO7 fluorescent signals by using Pearson correlation coefficient and the Mander’s 1 and 2 overlap coefficients, for the whole image and for single nuclei. **e** Functional interaction network analysis of 147 Pearson protein database using Cytoscape StringApp. Topological clustering algorithm showed functional interactions between groups of nodes. Pearson correlation index between CIN value, measured as wGII, and protein expression for the significant proteins were mapped to the nodes using a blue-white-red gradient. Proteins without any interacting partners within the network (singletons) and clusters of less than 4 nodes are omitted from the visualization. Each cluster was shaded following a colour code similar to the one used in the integration network according to their functional relationship. Cluster 1 related to metabolism is surrounded by an orange line and shading has been added only on mitochondrial proteins to highlight their predominance. The most enriched term per cluster was annotated. **f** Functional enrichment analysis of Pearson clusters expressed as enrichment fold in cyan blue. In magenta indicates the number of cluster proteins included in each term. A selection of those processes that best define the cluster proteins is shown in the figure. **g** Log2FC from RNAseq (left), proteomics data (centre) and Pearson's coefficient (right) for differentially expressed mitochondrial (Mit.) and non-mitochondrial (non-Mit.) proteins are plotted. Increased (red) and decreased (blue) protein values are indicated as a fraction. The *p*-value obtained by a hypergeometric distribution test is indicated. **h**-**i** Unsupervised Hierarchical Clustering Heatmap showing the Z-score protein expression values of mitochondrial proteins identified through the MitoCarta v. 3.0 across CIN- and CIN + PDOs and tissues dataset, pval < 0.0001 (Fisher-exact test)

To identify proteins and processes that best explain the CIN phenotype and could serve as biomarkers and/or therapeutic targets, we correlated organoid’ proteome with the wGII. We computed a protein-wGII Pearson correlation analysis and selected 147 proteins based on *p*-value (Additional file S3), 70 were in common with those of the integrated analysis while 77 where newly detected by Pearson (Fig. 5a). The data confirmed the relevance of laminins, showing a positive correlation with CIN (LAMA1: r 0·84, *p* < 0·001; LAMB1: r 0·802, *p* < 0·001; LAM C1: r 0·83, *p* < 0·001) as well as CDC42 (r 0·52, *p* < 0·05). In addition, we found new candidates not previously mentioned, such as IPO7 (r 0·53, *p* < 0·05), detected as differential at both proteomic and transcriptomic level (Fig. 3a), particularly interesting for its participation in the nuclear import of different proteins [41] including histone H1 and the protein component of human telomerase [42]. IPO7 dominant cargo is YAP, a key regulator of mechanotransduction, which is activated by CDC42 and once in the nucleus can activate the expression of Laminins and other EMT genes [43]. Although not detected by proteomics, *YAP1* mRNA levels were higher in CIN + PDOs (Fig. S9). On the other hand, we detected an enrichment of motifs recognised by TEAD transcriptional factors involved in YAP signalling (Fig. 4c). Taken together, these data strengthen a putative activation of YAP signalling in CIN + models. To support this hypothesis, colocalization analysis of IPO7 and YAP1 showed, not only a higher expression of both proteins in CIN + models (Fig. 5b-c), but also a significant correlation of their respective nuclear localization, supporting their putative association in CIN organoids (Fig. 5d). On the other hand, two members of the annexin A (ANXA) calcium-regulated phospholipid-binding protein family, with opposite correlations with CIN, presented the highest correlation: ANXA7 (r 0·91, *p* < 0·001) and ANXA1 (r −0·83, *p* < 0·001). ANXA7 was reported to promote EMT, contributing to hepatocellular carcinoma aggressiveness [44]. The role of ANXA1 is unclear, with contradictory reports [45] indicating it as either an inhibitor or an activator of EMT [46, 47]. Another highly correlated protein was the mitochondrial trifunctional enzyme subunit-alpha HADHA (r 0·82, *p* < 0·001) with a key role in FAO, again suggesting metabolism rewiring in the CIN phenotype.

Moving beyond individual proteins, a functional protein–protein interaction network was generated. A main principal network emerged, with two clear large modules related with ‘mitochondrial metabolism’ and ‘cytoskeleton and extracellular matrix’ and two small ones related with ‘endoplasmic reticulum (ER) stress response’ and ‘mRNA processing and translation’. Figure 5e shows the generation of eight clusters (shaded in different colours) with more than 4 nodes, with colour coding to indicate the degree of positive or negative Pearson correlation. Functional enrichment analysis of topological clustering (detailed in Fig. S10) was performed (Additional file S3) and selected processes that better define the proteins of the cluster are showed in Fig. 5f. Many clusters reinforced some of the principal features highlighted in the integrative analysis, but with noteworthy new findings. Cluster 1, related with mitochondrial metabolism, contained many proteins linked to acyltransferase activity and FAO, confirming their correlation with CIN but with two new acyltransferases: ACAA2 (r 0·73, *p* < 0·001), a rate-limiting enzyme catalysing the last step of the mitochondrial beta-oxidation pathway, and ACAT1 (r 0·61, *p* < 0·01), a key rate-limiting enzyme in ketone body metabolism responsible for recycling ketone bodies into acetyl-CoA, reinforcing a process already highlighted in the integrative analysis. As proof of concept, since previous studies indicated that increased acetyl-CoA coincides with elevated acetylation [48], we analysed lysine acetylation in proteins as it is the prevalent modification in chromatin, and observed a mostly nuclear signal, more intense in CIN + PDOs, although the difference is only significant in the model with the highest CIN value (Fig S11).

The TCA cycle and respiratory electron transport also correlated with CIN, with virtually the same proteins as in integration analysis plus citrate synthase (CS: r 0·59, *p* < 0·01), pyruvate dehydrogenase (PDHA1: r 0·49, *p* < 0·05) and ETFA (r 0·502, *p* < 0·05), a flavoprotein required for electron transfer to the respiratory chain from various acyl-CoA dehydrogenases involved in fatty acid and amino acid oxidation. Glutamine metabolism (with GLS1 and GLUD1: r 0·69, *p* < 0·01 and r 0·49, *p* < 0·05, respectively), previously undetected, was found to be correlated with CIN. Further supporting the role of mitocondrial function in CIN was the fact that among the differential proteins whose abundance increases, mitochondrial proteins are significantly over-represented, as can be observed in new Fig. 5g. Moreover, we analysed the expression levels of genes/proteins related with mitochondrial function using the MitoCarta 3.0 (https://www.broadinstitute.org/files/shared/metabolism/mitocarta/human.mitocarta3.0.html). Thanks to this approach we identified a set of proteins that are differentially expressed, showing that CIN + samples present a notably higher expression of mitochondrial proteins, as regards PDOs (Fig. 5h) as well as an independent CRC tissues dataset (Zhang et al.) (Fig. 5i). Taken together, these findings suggest an increased mitochondrial function in CIN + CRCs.

Cluster 2 highlighted the endoplasmic reticulum (ER) stress response, an adaptative survival mechanism activated by stressful circumstances, such as the unfolded proteins accumulation, exploited by cancer cells.

Regarding cell shape and stiffness, cluster 4 (enriched in the organization of extracellular matrix with laminins as prominent members) was detected, with a new protein, HSPG2 (r 0·69, *p* < 0·01), an extracellular matrix proteoglycan which contributes to invasion, metastasis, and angiogenesis in solid tumours, including CRC [49]. Furthermore, actin cytoskeleton organization was confirmed as an enriched process in clusters 5, 7 and 8, with new components. Among these, alpha- and beta-subunits of the heterodimeric CAPZ protein negatively correlated with CIN (CAPZA1: r −0·58, *p* < 0·05; CAPZA2: r −0·51, *p* < 0·05), while there was a positive correlation with CFL1 (r 0·48, *p* < 0·05), an essential regulator of actin filaments dynamics. The actin-capping protein CAPZ binds to the barbed end of actin, preventing actin filament growth [50]. Interestingly, CAPZA1 was also reported to inhibit EMT in hepatocellular carcinoma by regulating actin cytoskeleton [51]. In contrast, CFL1 was described as crucial in the switch from epithelial to mesenchymal-like morphology, and cell migration and invasion in CRC cells [52]. In the same line, keratins are the main identification markers of circulating tumours cells. In particular, KRT16 (r 0·68, *p* < 0·01) protein expression was associated with intermediate mesenchymal phenotype with a regulatory effect on EMT [53].

### Prioritisation of potential therapeutic targets associated with chromosomal instability in CRC

**Fig. 6:**
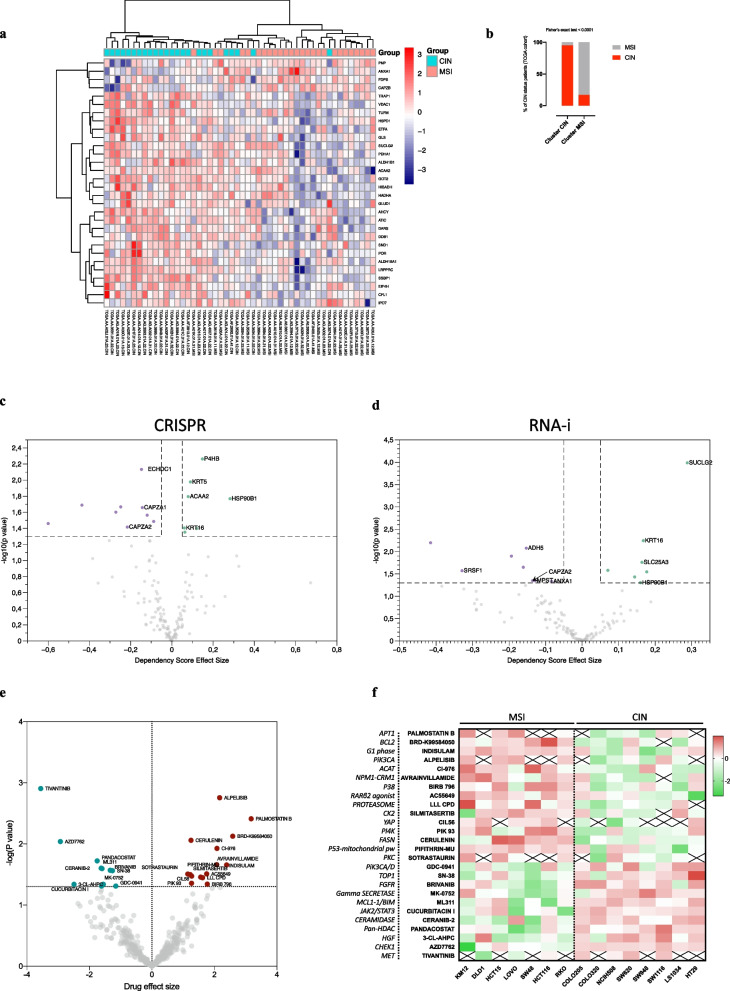
External validation of wGII related proteins and Cancer Dependencies analysis. **a** Unsupervised Hierarchical Clustering Heatmap showing the 30 differentially expressed proteins between CIN and MSI tissues (as proxies of CIN-) which are concordant with our cohort in an external independent proteomic cohort. The colour code indicates Z-score of protein abundance. The columns corresponding to each sample are indicated at the bottom while its corresponding CIN/MSI status is colour-coded above. **b** Fisher-exact test CIN/MSI cohort vs clustering based on the 30 concordant proteins. **c-d** Volcano plot representation of dependency score effect in CIN + versus CIN- CRC cell lines using CRISPR and RNA-interference screening, respectively. Genes with significantly increased or decreased dependency score (*p*-value < 0·05) are differently coloured. Genes concordant with Pearson analysis are marked with their names. **e** Volcano plot representation of drug effect size in CIN + versus CIN- CRC cell lines. Drugs with significantly increased or decreased effect (*p*-value < 0·05) are differently coloured and marked with their names. **f** Z-score Ln-AUCs heatmap (red: no response; green: good response) of drugs with a significant effect. Left column: target of each compound

To prioritise proteins or processes found associated with CIN in our organoids we leveraged publicly available databases. We first evaluated the expression of wGII-protein list across an independent proteomic dataset [18] where protein abundance was determined in a similar manner, although CIN was transcriptomically defined. We found that 44 of 147 proteins of our list were differentially expressed between CIN and MSI tissues (Additional file S4), with 30 showing the same change in expression as in our organoid cohort, including, for instance, IPO7, CFL1, LRPPRC, ANXA1, SUCLG2, ACAA2 and HADHA, the last three being acyltransferase enzymes. The acyltransferase ACAT1 was not significantly differentially expressed, but we found a trend towards an increase in its expression in CIN tissues (Fig. S12). Unsupervised clustering heatmap showed that these proteins were able to classify the tissues according to CIN (Fisher exact test, *p*-value < 0·0001) (Fig. 6a-b).

Next employing functional genomics databases (DepMap) we analysed the role of wGII-proteins in CIN through loss-of-function screens in 15 CRC cell lines compatible with CIN and MSI status [54]. For RNA-i not all genes were present, so we analysed the effect of 130 of them (Supplementary Methods and Additional file S4). Among the genes with stronger dependencies in CIN + cell lines (Fig. 6c-d) seven encode proteins positively correlated with CIN: two chaperones, related to ‘ER stress response’, HSP90 and P4HB; two mitochondrial enzymes, SUCLG2 and ACAA2, linked to acyl-CoA transference in TCA and FAO metabolic pathways respectively; the mitochondrial phosphate carrier SLC25A3; and two keratins, KRT5 and KRT16, cytoskeleton structural proteins.

We also queried DepMap to determine in a large-scale screening those drugs, among the 497 checked, that were more effective in CIN + cell lines (Additional file S4). After eliminating compounds without a defined target and those for whom only one line was tested, we identified 26 of them with a differential effect, 15 significantly more effective in CIN + with respect to MSI cell lines (Fig. 6e). Two of them caught our attention: the YAP inhibitor CIL56, since our data point to a possible activation of YAP signalling in our models, and CI-976, an inhibitor of cholesterol acyl transferase (ACAT1/SOAT1).

Thereafter, we aimed at analysing the prognostic effect of prioritised targets using KM-plot, considering MSS patients as a good approximation of CIN patients, as CIN status was not available and as an inverse relation has been observed between CIN and MSI [55, 56]. Interestingly, we found that, some targets influenced survival. These included, YAP1, IPO7, CDC42, LAMB1, LAMC1, KRT16 and GLS higher expression was associated with lower survival in MSS patients (Fig. S13) (OS: YAP1 HR 1·7, pval = 0·000088; IPO7 HR 1·37, pval = 0·059; CDC42 HR 1·73, pval = 3e-04; LAMB1 HR 2·39, pval = 0·00081; LAMC1 HR 2·37, pval = 0·00052, KRT16 HR 1·58, pval = 0·0038; GLS HR 1·67, pval = 0·00083), while for ACAT1 there was an effect on relapse-free survival in advanced stages (1·51, pval = 0·027) (Fig. S14). Furthermore, all these survival effects were specific for MSS patients as in MSI there was either no significant effect or the opposite effect, as in the case of CDC42 and ACAT1 (Fig. S13-S14). Finally, we analysed the expression of these prioritised targets in CRC versus matched normal mucosa using the TNM-plot tool (https://tnmplot.com/analysis/) and we found that most of them show tumour-specific mRNA expression except for ACAT1 and CDC42, in contrast to the literature [31, 57] (Fig. S15).

Table 1 summarizes the prioritised processes and targets identified.

**Table 1:** Prioritised processes and proteins identified as possible targets in CIN CRC

PPI network Functional Modules	ENRICHED term	Prioritised Proteins	Correlation	CRISPR/RNAi dependence	Validation in external cohort	Survival effect	Hypothetical Role	References
Extracellular matrix and Cytoskeleton	Actin cytoskeleton organization	CDC42	Positive			✓	IPO7 activation	[42]
	Actin cytoskeleton organization	CFL1	Positive		✓	✓	Switch from epithelial to mesenchymal-like morphology	[50]
	Extracellular matrix glycoproteins	LAMB1	Positive			✓	EMT YAP positive feedback	[35, 36, 78]
		LAMC1				✓		
		LAMA1						
	Protein nuclear import	IPO7	Positive		✓	✓	YAP import	[41, 42]
	Keratin	KRT16	Positive	✓		✓	EMT regulation	[51]
	Actin cytoskeleton organization	CAPZA1	Negative	✓			EMT inhibition	[48, 49]
		CAPZA2		✓				
	Signaling receptor binding	ANXA1	Negative	✓	✓		EMT inhibition	[46]
Mitochondrial metabolism	FAO/ acyltransferase activity	HADHA	Positive	✓	✓		Acyloma remodeling Mitochondrial energy metabolism rewiring	[38, 39, 58]
		ACAA2			✓			
	Ketone body metabolism/ acyltransferase activity	ACAT1	Positive			✓	Acyloma remodeling Reutilization of ketone bodies to fuel mitochondrial metabolism	[53, 69, 70]
	TCA cycle/ acyltransferase activity	SUCLA2	Positive	✓	✓		Acyloma remodeling Metabolism rewiring	[59–61]
		SUCLG2						
	Respiratory electron transport	PDHA1	Positive		✓		Metabolism rewiring	[38, 57]
		ETFA			✓			
	Glutamine metabolism	GLS1	Positive		✓	✓	Glutamine conversion to fuel TCA cycle EMT induction	[39, 71–73]
		GLUD1			✓			
	ATP synthasome	SLC25A3	Positive	✓	✓		Mitochondrial ATP synthesis	[29]
ER stress	Protein folding	HSP90B1	Positive	✓			Response to ER stress/Chaperone	[57]
		P4HB		✓				

The table shows the CIN correlation sign (positive/negative), the validation in different public databases (✓ symbol) and the hypothetical role in CIN PDOs for each target

## Discussion

**Fig. 7:**
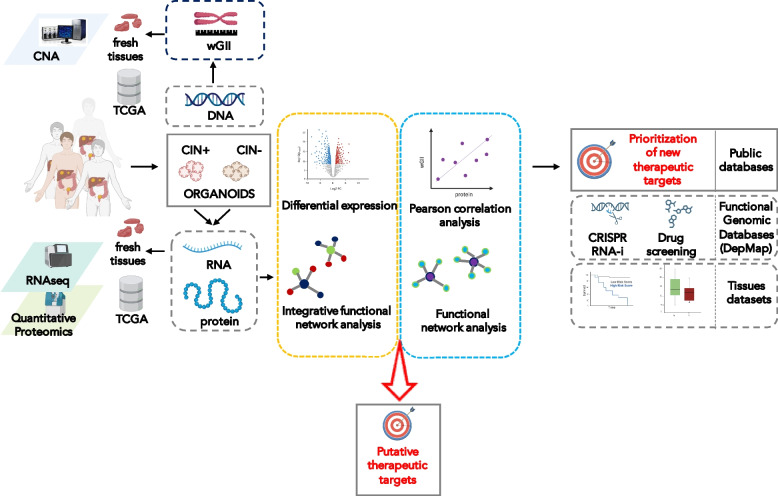
Study workflow

This work sheds light on the mechanisms operating in advanced CRC CIN tumours. We demonstrated that PDOs faithfully reproduced the genome, transcriptome and proteome of tissues across independent datasets. Integration of differential RNA and protein expression data of CIN + vs CIN- PDOs allowed us to identify functional modules. Then, we correlated the proteome with the CIN value, highlighting the proteins that best explain CIN phenotype. Finally, using functional genomic databases and patient-tissues datasets we prioritized, in silico, some of the high-confidence CIN features of organoids to be explored in future functional studies. Figure 7 illustrates the experimental workflow.

To understand the molecular mechanisms underlying CIN, the use of models as much representative as possible of human tumours is imperative. Recent data addressed CIN in organoids at genome level [12]. We also integrated it with a deep RNA and protein expression characterization. First, we determined wGII [17, 58] as a proxy for CIN in organoids and tissues, showing a significant concordance and dynamic increase, as proven by patient 24. Although CIN generation and tolerance represents an important bottleneck during tumour progression[59, 60], the ability of chromosomal unstable cells to generate genomic heterogeneity in their progeny allows the tumour to evolve and progress. Accordingly, organoids captured inter and intrapatient heterogeneity.

Starting from the most differentially expressed genes in organoids, we were able to classify TCGA tissues, showing that our models were a good phenotypic reproduction of CIN in CRC (Fig. 2d). The wider effects of CIN on the transcriptome are poorly understood, and even fewer studies addressed its impact on the proteome. Our integrated proteotranscriptomic analysis (Fig. 3) enhanced the understanding of the expression patterns associated with CIN in PDOs.

Alterations in gene content cause significant energy and proteotoxic stress impairing cell fitness [61]. Accordingly, functional enrichment identified mitochondrial metabolism comprising FAO, TCA cycle and OXPHOS in CIN models. Moreover, mitochondria generate metabolites involved in survival, growth, and gene expression regulation [62]. Indeed, we observed changes in the expression of TCA cycle enzymes involved in the production of oncometabolites that control chromatin epigenetic changes and proteins PTMs (as IDH3A, OGDHL, SUCLA2, SUCLG1 and SUCLG2). Alterations in SUCLA2 expression led to changes in succinyl-CoA levels and global protein succinylation, regulating different mitochondrial metabolic networks like the TCA cycle flux [63] and contributing to different diseases including cancer [64, 65]. Other processes enhanced in CIN would contribute to increasing the cellular pool of acyl-CoA species, such as BCAA, FAO and ketone body metabolic pathways [66]. Recent evidence indicate that nucleus and mitochondria maintain a bidirectional regulation and through “retrograde signaling” mitochondria can regulate the expression of different genes to control cell fate and function [67]

Other relevant features associated with CIN were related to ‘extracellular matrix and cytoskeleton organization’, with a clear upregulation of genes involved in EMT and a significant reduction of the epithelial marker EpCAM, one of the most consistently downregulated proteins associated with EMT phenotype in different models [65]. Indeed, we identified a prominent CIN-associated cluster consisting of four laminins, implicated in EMT and aggressive cancer phenotype [68, 69]. Additionally, we observed a notably higher expression of the EMT/stem marker *CD44* in CIN PDOs. Interestingly, another EMT inducer *Twist1* was shown to downregulate cell cycle checkpoints involved in genome stability [70] and CIN-high PDOs were successfully classified using the transcriptomic EMT signature (Fig. 4), as previously reported with CIN cancer cell lines [71]. Motif analysis showed a relevant activity of transcription factors reported to induce EMT, as well as some involved in mitochondrial function. Furthermore, cells undergoing EMT show metabolic changes to balance proliferation versus energy-consuming migration, indicating a crosstalk between EMT and metabolic reprogramming [39, 40, 72].

CIN, as a dynamic process with increasing genomic imbalance, is more accurately characterized as a “rate” rather than a cellular “state”. On the other hand, although CIN shows significant effects on mRNAs abundance, relatively few changes extend to the protein level [18]. Taking these two issues into account, correlation analysis between the degree of CIN (measured as wGII) and the level of protein expression could help to identify candidate driver genes and processes in CIN. A new functional network based on the list of proteins with significant Pearson correlation index was constructed (Fig. 5) and GO terms reinforced an enrichment of the ‘mitochondrial metabolism’ with a key role of FAO, as well as ‘extracellular matrix’ with laminins and ‘cytoskeleton organization’ and, a new module related to ‘endoplasmic reticulum (ER) stress response’ one of the most important mechanisms regulating cellular adaptation to adverse cellular conditions, including aneuploidies [8]. In addition, new proteins appeared, notably ACAT1, a key enzyme that allows ketone body re-utilization into acetyl-CoA. Previous studies established that ketone bodies fuel mitochondrial activity, leading to an increase in ATP production in cancer cells [73, 74], actively promoting tumour growth and metastasis. However, ketone bodies can also act as signalling metabolites, influencing gene expression and PTMs, among other processes. Recently, it was shown that elevated serum β-hydroxybutyrate, a circulating ketone metabolite, accelerates CRC proliferation and metastasis via ACAT1 due to the induction of IDH1 acetylation [57]. Other emerging proteins were GLS1 and GLUD1, involved in glutaminolysis, the process by which glutamine is converted into TCA cycle metabolites. GLS1 is overexpressed in various cancer cells associated with poor prognosis [75] and both were described as EMT inducers in cancer cells [76, 77].

The correlation analysis reinforced the EMT phenotype in CIN PDOs. Indeed, the most positively correlated proteins include laminins, which activate the GTPase CDC42 [78], itself with a positive correlation. It is involved in the modification of actin cytoskeleton, which is known to activate YAP, connecting nuclear import processes with mechanical extracellular cues and actin cytoskeleton [79]. A YAP activated signature was shown to predict poor outcomes in patients with CRC [80] and this aligns with findings associating high YAP expression and nuclear localization with adverse patient outcomes [81]. Intriguingly, the importin IPO7 was positively correlated with CIN, with YAP its principal cargo. Nuclear YAP can induce the expression of laminins and other EMT-related genes, establishing positive feedback [82]. Although little is known about IPO7 cellular function, this gene is frequently overexpressed in CRC, induced by c-MYC and downregulated by p53 [83]. We showed an increase in active YAP and IPO7 expression and their significant colocalization in CIN + organoids, reinforcing a putative IPO7/YAP axis leading to its activation. If confirmed, this could represent a new putative target for drug development [84] in the context of CIN.

To prioritize among the proteome-wGII list, we used a publicly data-driven approach. Since MS-based proteomics has not been implemented in clinical practice, there are not many studies containing both proteome and clinical data. By using the data available at Zhang B. et al*.* [18], we found that 30 proteins were able to classify CIN tumours in this cohort, including IPO7, SUCLG2, ACAA2, and HADHA (Fig. 6a). Furthermore, the interrogation of functional genomic databases revealed some proteins as genetically dependent in CIN cells (Fig. 6c-d). Two chaperones, HSP90B and P4HB, related with ‘ER stress response’ and, again, two mitochondrial acyl-CoA transferases, ACAA2 and SUCLG2, involved in FAO and TCA. Moreover, drug sensitivity from DepMap pointed to the inhibition of YAP and acyltransferase processes as the most effective targets in CIN + cell lines (Fig. 6e-f). Finally, despite CIN status was not available and the expression measured at mRNA level, higher gene expression of *IPO7*, *YAP1, CDC42, LAMB1, LAMC1, KRT16, GLS* and *ACAT1* is associated with lower survival in MSS but not in MSI patients (Fig. S13-14).

There are some limitations in this work that require further studies, starting from in vitro or in vivo mechanistic experiments, necessary to confirm the role of the multiple candidates provided by our study, as putative Achilles’ heels in CIN tumours. In addition, due to the intrinsic epithelial nature of PDOs, we were unable to detect signs of immune involvement, such as cGAS-STING pathway [85]. In addition, the intrinsic limit of our proteomics approach that only captures peptides with canonical sequences, does not allow to capture neoantigens which CIN could generate.

In summary, unlike cancer cells, normal cells cannot tolerate errors in chromosome segregation. Understanding how cancer cells cope with the deleterious consequences of CIN could open new therapeutic opportunity. We show the utility of organoids to study CIN in CRC as they recapitulated the genomic and phenotypic features of CIN. The validation of all omics data in independent tissue cohorts in the context of CIN strengthens the generalisability of our findings. The expression patterns identified herein should serve as a useful ex vivo marker for cancer progression and could be exploited to develop new therapeutic strategies selectively targeting high-CIN cells. Furthermore, the primary and processed datasets generated herein could be used for new biological discoveries and therapeutic hypotheses generation.

## Conclusions

In conclusion, we demonstrated that PDOs from advanced CRC patients are a good in vitro model of chromosomal instability in terms of genomics, transcriptomics and proteomics. Our findings identify new putative targets that could be exploited in the future to develop new therapeutic strategies selectively targeting high-CIN cells.

## Supplementary Information

Supplementary Material 1: Supplementary methods. Table S1. Reagents and tools. Table S2. Patient characteristics. Fig. S1. Whole genome heatmap representation of copy number gains (red) and losses (blue) across all tissues and PDOs. Fig. S2. Venn diagrams of gene-level copy number alterations depicting subclonal inter-metastasis heterogeneity. Fig. S3. (a) Hematoxilin and eosin staining of representative PDOs showing some examples of atypical mitotic figures in CIN- (A-B) and CIN+ (C-D) PDOs. (b) Heatmap representing the mutational and copy number status of PDOs. Fig. S4. GSEA analysis of CIN+ vs CIN- PDOs. qvalue threshold is set at <0.05. Fig. S5. Chi-square CIN+/- cohort vs clustering based on top 100 gene signature. Fig. S6. Unsupervised hierarchical clustering heatmaps of PDOs Z-score gene expression across different CIN signatures: CIN70 and HET70. Fig S7. Correlation analysis between RNA and protein fold change for those detected as differential by both RNAseq and proteomics (red dots) and those differential at RNA level detected by proteomics. Fig S8. (a) Activity Score by mean of Z-value obtained by ISMARA, sorted by Z-value higher than 1.3. (b) ISMARA transcription-factor- activity plot of the CIN- and CIN+ models. n = 3 biological replicates. Fig. S9. violin plot indicating YAP1 expression level in CIN+ vs CIN- organoids. n=3 biological replicates. Fig. S10. Clustered protein association network derived from Pearson correlation analysis. Clustering was performed using the Markov clustering implementation in the Cytoscape-StringApp. Fig. S11. (a) Confocal imaging of CIN- (upper panel) and CIN+ (lower panel) organoids stained with DAPI (blue) and anti-Acetylated-Lysine (red). Representative images are shown. (b) Single nuclei staining intensity quantification for Acatylated-Lysine. Fig. S12. Protein abundance of ACAT1 across MSI and CIN tissues. Fig. S13. Target’s expression is associated with an impact on OS. Representation of OS Kaplan Meier curves in MSS and MSI-CRC samples from TCGA based on an optimal cut-off. Fig. S14. Target’s expression is associated with an impact on RFS. Representation of RFS Kaplan Meier curves in MSS and MSI-CRC samples from TCGA based on an optimal cut-off. Fig. S15. Box plots of YAP1 and IPO7 gene expression in the TCGA (tumour versus normal mucosa near the tumour area).

Supplementary Material 2. Additional File S1. Excel file containing organoids and tissues copy number data.

Supplementary Material 3. Additional File S2. Excel file containing organoids gene signature deriving from transcriptomic analysis used to classify TCGA tissues and differential gene expression analysis.

Supplementary Material 4. Additional File S3. Excel file containing proteomic dataset and full results of integrative and Pearson analysis.

Supplementary Material 5. Additional File S4. Excel file containing public databases analysis.

## Data Availability

This study did not generate new unique reagents. PDOs models are stored in the INCLIVA Biobank. Cytoscan HD and sequencing data have been deposited in ENA repository. The mass spectrometry proteomics data have been deposited to the ProteomeXchange Consortium via the PRIDE partner repository. Accession numbers are listed in the reagents and resources table. All copy number, DEseq and enrichment are provided as supplementary material.

## Abbreviations

BCAA: Branched-chain amino acids
CIN: Chromosomal instability
CNA: Copy number alterations
CRC: Colorectal cancer
EMT: Epithelial-mesenchymal transition
ER: Endoplasmic reticulum
FAO: Fatty acid beta-oxidation
GOs: Gene ontologies
PDOs: Patient-derived organoids
PTMs: Post-translational modifications
TCA: Tricarboxylic acid cycle
wGII: Weighted Genome Instability Index
