# Supplementary material for: Decoding chromosomal instability insights in CRC by integrating omics and patient-derived organoids
Source: J Exp Clin Cancer Res. 2025 Feb 28;44:77. doi: 10.1186/s13046-025-03308-8 (PMC11869439; doi:10.1186/s13046-025-03308-8)
Supplement: Supplementary file 1 — Supplementary Material 1: Supplementary methods. Table S1. Reagents and tools. Table S2. Patient characteristics. Fig. S1. Whole genome heatmap representation of copy number gains (red) and losses (blue) across all tissues and PDOs. Fig. S2. Venn diagrams of gene-level copy number alterations depicting subclonal inter-metastasis heterogeneity. Fig. S3. (a) Hematoxilin and eosin staining of representative PDOs showing some examples of atypical mitotic figures in CIN- (A-B) and CIN+ (C-D) PDOs. (b) Heatmap representing the mutational and copy number status of PDOs. Fig. S4. GSEA analysis of CIN+ vs CIN- PDOs. qvalue threshold is set at <0.05. Fig. S5. Chi-square CIN+/- cohort vs clustering based on top 100 gene signature. Fig. S6. Unsupervised hierarchical clustering heatmaps of PDOs Z-score gene expression across different CIN signatures: CIN70 and HET70. Fig S7. Correlation analysis between RNA and protein fold change for those detected as differential by both RNAseq and proteomics (red dots) and those differential at RNA level detected by proteomics. Fig S8. (a) Activity Score by mean of Z-value obtained by ISMARA, sorted by Z-value higher than 1.3. (b) ISMARA transcription-factor- activity plot of the CIN- and CIN+ models. n = 3 biological replicates. Fig. S9. violin plot indicating YAP1 expression level in CIN+ vs CIN- organoids. n=3 biological replicates. Fig. S10. Clustered protein association network derived from Pearson correlation analysis. Clustering was performed using the Markov clustering implementation in the Cytoscape-StringApp. Fig. S11. (a) Confocal imaging of CIN- (upper panel) and CIN+ (lower panel) organoids stained with DAPI (blue) and anti-Acetylated-Lysine (red). Representative images are shown. (b) Single nuclei staining intensity quantification for Acatylated-Lysine. Fig. S12. Protein abundance of ACAT1 across MSI and CIN tissues. Fig. S13. Target’s expression is associated with an impact on OS. Representation of OS Kaplan Meier curves in MSS [file 13046_2025_3308_MOESM1_ESM.pdf]

SUPPLEMENTARY METHODS

Ethics

The study was approved by the Hospital Clínico Universitario de Valencia Ethics Committee (2018/063, 2021/083). All patients provided written informed consent for the collection of fresh tissues and clinical data. The study followed all approved protocols and procedure and was conducted in compliance with the Declaration of Helsinki.

Tissue processing and organoid culture

Fresh tissues were collected in PBS with penicillin/streptomycin and quickly processed as previously published[9]. Briefly, tissue samples were minced into small fragments. Some were stored for DNA and RNA extraction depending on the total amount of tissue. All the remaining fragments were mechanically and enzymatically digested with a collagenase-based solution (Sigma- Aldrich, cat. No. 269395) until reaching single cells. These were resuspended in basal media with 50% reduced-growth factor basement membrane matrix (BME Type 2, R&D, cat. No. 3533-010-002) and plated in prewarmed culture plates in complete medium with the following composition: 1x N2 (cat.no. 17502048), 1x B27 (cat.no. C21-H), 1mM N-acetyl L-cysteine (cat.no. A9165), 10 mM nicotinamide (cat.no. N0636), 50 ng/mL hr-EGF (cat.no. BMS320), 100 ng/mL hr-noggin (cat.no. 6057-NG-025), 0·5 µM A-83-01 (cat.no. SML0788), 10 µM SB202190 (cat.no. S7067), 10 nM gastrin (cat.no. G9145), 500 ng/mL hr-RSPO1 (cat.no. 4645- RS), 10 ng/mL hr-FGF10 (cat.no. 100-26), 10 nM PGE2 (cat.no. P6532), 10 µM Y-27632 (cat.no. Y0503). After reaching the appropriate volume, organoids were trypsinized with TrypLE™ Express (Life Technologies, cat. No. 12605-010) to expand them. Aliquots were stored in liquid nitrogen to constitute a biobank. Supplementary table S2 shows the main features of the organoids models employed in this paper and the kind of analysis performed for each one.

Copy number analysis

Fresh tissues and PDOs DNA were extracted with the QIAamp DNA Micro Kit (Qiagen, cat. No. 56304). Cytoscan HD for whole genome copy number variation was performed according to manufacturer protocol in the Multigenic Analysis Service (UCIM), Faculty of Medicine, Valencia. Briefly, between 80 and 500 ng of integer genomic DNA was digested and ligated. Amplicons were generated by performing PCR using primers provided by the manufacturer (Affymetrix, CA) using the following program: 94°C for 3 min, then 35 cycles of 94°C 30 sec, 60°C for 45 sec and 65°C for 1 min. This was followed by extension at 68°C for 7 min. The PCR products were then purified and digested with DNAaseI for 35 min at 37°C to fragment the amplified DNA. The fragmented DNA was then labeled with biotinylated nucleotides through terminal deoxynucleotide transferase for 4 hours at 37°C. Fragmented DNA was hybridized with a pre-equilibrated Affymetrix chip Cytoscan HD chip at 50°C for 18 hours. CytoscanHD chips washing and scanning followed the procedures of the Affymetrix Inc. manuals. CEL files were generated from AGCC software from Affymetrix, Inc. (Santa Clara, CA).

CytoScan HD CEL files were processed through Chromosome Analysis Suite (ChAS) software version 4.1 with a single sample algorithm. All samples were manually reviewed, and unbalanced samples were reprocessed with normal diploid algorithm. Obtained Chyp files were analyzed with ChAS and IGV software v. 3.0. Copy number data were retrieved for further analysis.

CIN status determination

Weighted Genome Instability Index (wGII) was calculated as published elsewhere[10]. Briefly, genomic co-ordinates of all gain and lost regions were retrieved from ChAS software for each sample. Co-ordinates were manually reviewed to exclude overlap between regions. The total of copy number alterations (CNA) in terms of base pair (bp) was calculated and normalized for chromosome length and wGII expressed as median CNA across the genome. wGII correlation between PDOs and matched fresh tissue was conducted with linear regression analysis. All the calculations were conducted in RStudio.

Hematoxylin and eosin staining

As previously described [9], PDOs domes were collected in 4% neutral buffered formalin and paraffin embedded. 4 um slides were cut, dewaxed and hematoxylin and eosin (Dako, cat. No. CS700 and CS701) staining was performed. Mitotic figures count has been performed by an expert pathologist.

RNA-Sequencing (RNA-seq)

As previously published [9], total RNA was extracted from fresh tissues and PDOs with RNeasy Micro Kit (cat. no. 74004) and integrity verified with TapeStation RNA Analysis ScreenTape (Agilent Technologies). Sequencing libraries were prepared with the NEBNext® Ultra (TM) II Directional RNA Library Prep Kit for Illumina® Module (New England BioLabs) and NEBNext Poly(A) mRNA Magnetic Isolation Module for mRNA enrichment following the manufacturer's instructions. Libraries were paired end sequenced in an Illumina NextSeq 550 platform with a NextSeq 500/550 High Output 300 cycles kit.

Proteomics analysis by LC-MS/MS-SWATH

Sample preparation

As previously described[9], PDO pellets were lysed in UTC buffer (8M urea, 2M thiourea and 4% CHAPS) and the protein concentration measured with the RC DC Protein Assay (Bio-Rad). A pooled sample containing 5 µg of protein from each sample was used to prepare the peptide library. For the SWATH analysis, 20 µg of protein from each sample were used. Samples were denatured at 95 °C for 5 min in sample buffer and subjected to sodium dodecyl sulfate-polyacrylamide gel electrophoresis (SDS-PAGE). Pooled samples were resolved in gel and lanes were cut into five pieces. Gels containing individual samples were not resolved and whole samples were sliced into a single piece. Protein digestion and subsequent analysis were performed as published elsewhere (Pinheiro et al., 2020) at the Proteomics Service (SCSIE) of the University of Valencia.

LC-MS/MS

Spectral peptide library was obtained by liquid chromatography and tandem mass spectrometry (LC-MS/MS) analysis, operating the instrument in a data-dependent acquisition mode. Peptide mixtures were loaded onto a trap column (3µ C18-CL, 350 µm x 0·5mm; Eksigent) and desalted with 0·1 % TFA at 5 µL/min for 5 min. Peptides were then loaded onto an analytical column (3µ C18-CL 120 Ă, 0·075 x 150 mm; Eksigent) equilibrated in 5% acetonitrile 0·1 % FA (formic acid). Elution was carried out with a linear gradient of 7-40 % buffer B in A (A: 0·1 % FA; B: 5 % acetonitrile (ACN), 0·1 % FA) at a flow rate of 300 nL/min over 60 min. Eluted peptides were analyzed in a mass spectrometer nanoESI-qQTOF (TripleTOF 6600; SCIEX). Up to 100 ions were selected for fragmentation after each survey scan. Data files were processed using the ProteinPilot search engine (version 5.0, SCIEX) to search the Swiss-Prot database with the following parameters: Trypsin specificity, IAM cys-alkylation, Species *Homo sapiens*, and the search effort set to thorough and FDR correction.

Quantitative analysis of individual samples was performed by sequential window acquisition of all theoretical spectra-mass spectrometry (SWATH-MS). Peptides were analyzed by LC using a trap column and analytical column as previously described but operating the TripleTOF 6600 mass spectrometer instrument in SWATH mode. We used 100 variable windows from 400 to 1250 m/z with a total cycle time of 2·79 seconds. Quantitative data was extracted from .wiff files with Peak View 2.2 (SCIEX) using the peptide library generated by ProteinPilot as indicated above. For every protein in the spectral library, a maximum of 20 peptides were quantified among those with a confidence threshold of 95 % and a false discovery rate (FDR) lower than 1 %. Shared peptides were excluded. For every peptide, a maximum of six transitions (fragment ions) were quantified. Biological quadruplicates were employed.

Data analysis

For RNA-seq data, read quality control was performed with FASTQC v0.11.8. Sequencing adapters and low-quality reads were filtered out with fastp v0.11.8. Filtered reads were mapped against the human reference genome GRCh38 using STAR v2.7.3a. Isoform quantification was performed with RSEM v1.3.3 and further processed with Tximport v1.16.1. Differential expression analysis was conducted with the DESeq2 v1.34 package with RStudio. Resulting p-values were adjusted with the Bonferroni method. Significant genes were selected based on an adjusted p-value cutoff of 0·01 and an absolute log-folchange greater than 1. GSEA analysis was run against the "Hallmark" gen set from the MSigDB. The significance threshold was set at P-value below 0.05 and nominal FDR below 0.05. The "EMT" gene set (Hallmark_epithelial_mesenchymal_transition) from the Molecular Signatures Database (MSigDB, GSEA) was used to

explore de EMT phenotype across the organoid lines. Unsupervised hierarchical cluster analysis was done using a Euclidean distance measure and Ward linkage with the RStudio.

Motif analysis has been conducted using FASTQ files to run ISMARA software at (https://ismara.unibas.ch/mara/). Obtained motif z-values have been filtered out excluding those lower than 1.5.

SWATH quantitative data (protein areas) were median normalized and log2 transformed. Differentially expressed proteins were selected by Student t-test using a p-value of 0·05. Functional analysis of differential proteins was performed using STRING database and Cytoscape StringApp within Cystoscape software.

The correlation between CIN and protein abundance was analyzed by calculating the Pearson coefficient. We used the Pearson function of Microsoft Excel spreadsheet with the median normalized and log2 transformed data for each protein and the wGII value for each sample as function inputs.

Statistical analyses

The significance threshold of all stastistical analyses was set at p-value below 0.05. wGII correlation between PDOs and matched fresh tissue was conducted with linear regression analysis. The PDOs-TCGA transcriptomic comparison was performed with Chi-square test. The correlation between CIN and protein abundance was analyzed by calculating the Pearson coefficient. We used the Pearson function of Microsoft Excel spreadsheet with the median normalized and log2 transformed data for each protein and the wGII value for each sample as function inputs. Venn diagrams were used to visualize differences between categorical groups. Statistical analyses related to publicly datasets are described in the corresponding section.

Publicly available datasets analysis

For cell lines dependencies analysis, we selected CIN+ and CIN- cell lines and downloaded combined CRISPR/Cas9 DepMap Public 23Q4+Score and Chronos datasets and from RNA-interference combined Achilles+DRIVE+Marcotte, DEMETER2 from DepMap portal (https://depmap.org/portal/) selecting genes from the Pearson signature proteins. Not all corresponding genes were present in the RNA-interference datasets, therefore in this case the analysis was conducted on 130 genes. We compared the dependence score for each gene in CIN+ with respect to CIN- via multiple t-test. Data were represented with Volcano plots indicating the dependence score effect size.

For drugs analysis we downloaded Drug sensitivity AUCs from CTD^2 dataset from DepMap portal. Data belonging to compounds without a defined target and those for whom only one line was tested for each comparison group (CIN+ and CIN-) were discarded. Therefore, data related to 379 drugs were analyzed. We compared the AUCs for each compound in CIN+ with respect to CIN- via multiple t-test. For each drug a pattern was assembled containing the corresponding Ln-AUCs across the cell lines. Next, Z-scores were calculated for each drug, taking into account mean Ln-AUC and standard deviation for each point (LnAUC-mean/standard deviation). Finally, the patterns were aggregated column-wise into a matrix. The obtained matrix was used to assess the relative sensitivity/resistance of each line.

For proteomic public dataset we downloaded proteomic data from Zhang et al [48] and for CIN and MSI annotated samples selected those proteins that we identified from the Pearson signature. We compared protein abundance for each gene in CIN+ with respect to CIN- via multiple t-test. Furthermore, for each (significant) protein a pattern was assembled containing the corresponding across the tissue samples. Next, Z-scores were calculated for each protein, taking into account mean protein abundance and standard deviation for each point (protein abundance-mean/standard deviation). Finally, the patterns were aggregated column-wise into a matrix. Clustering heatmap was built with pheatmap package (Rstudio). Fisher exact test was used to evaluate statistical significance of tissue categorization.

Kaplan-Meier plotter software (https://kmplot.com/analysis/) (1-2) was used to evaluate the prognostic value of the prioritized targets. OS was analyzed based on the expression of CRC patients from the TCGA group, MSS and MSI cohort with auto- selected best-cut-off. The Hazard ratio (HR) and log- rank P-value are calculated by the software and curves are plotted.

We used the TNMplot web tool (https://tnmplot.com/analysis/) (3) to compare gene expression in CRC tumor versus normal tissue using RNAseq data and normal tissues near the tumor area. Data are represented as box plots.

Supplementary References

1. Lánczky A, Győrffy B. Web-Based Survival Analysis Tool Tailored for Medical Research (KMplot): Development and Implementation. J Med Internet Res. 2021 Jul 26;23(7):e27633. doi: 10.2196/27633
2. Győrffy B: Integrated Analysis of Public Datasets for the Discovery and Validation of Survival-Associated Genes in Solid Tumors, The Innovation, 2024, DOI: 10.1016/j.xinn.2024.100625
3. Bartha Á, Győrffy B. TNMplot.com: A Web Tool for the Comparison of Gene Expression in Normal, Tumor and Metastatic Tissues. Int J Mol Sci. 2021 Mar 5;22(5):2622. doi: 10.3390/ijms22052622

SUPPLEMENTARY TABLE

Table S1 REAGENTS AND TOOLS TABLE

Reagent/Resource	Reference or Source	Identifier or Catalog Number
**Experimental Models**		
Human colorectal cancer tissues	This paper	N/A
Patient-derived organoids	This paper	N/A
**Chemicals, peptides, and recombinant proteins**		
N-2 supplement (100X)	ThermoFisher Scientific	17502048
B27 Supplement (NCS21 Supplement (50x), Serum-free)	Labclinics	C21-H
N-acetyl L-cysteine	Sigma-Aldrich	A9165
Nicotinamide	Sigma-Aldrich	N0636
Human Recombinant EGF	eBioscience	BMS320
Human Recombinant Noggin	R&D systems	6057-NG
A-83-01	Sigma-Aldrich	SML0788
SB202190	Sigma-Aldrich	S7067
[Leu15]-Gastrin I human	Sigma-Aldrich	G9145
Human Recombinant RSpondin 1	R&D systems	4645-RS
Human Recombinant FGF-10	Peprotech	100-26
Prostaglandin E2	Sigma - Aldrich	P6532
Y-27632	Sigma - Aldrich	Y0503
TrypLE™ Express	Life Technologies	12605-010
BME Type 2	R&D	3533-010-002
DAPI	Invitrogen	P36941
YAP1 antibody	Abcam	ab205270
IPO7 antibody	Santa Cruz acetylated-lysine	sc-365231
Acetylated-Lysine antibody	Cell signalling	9441
**Critical commercial assays**		
QIAamp DNA Micro Kit	Qiagen	56304
RNeasy Micro Kit	Qiagen	74004
CytoScan™ HD Array Kit and Reagent Kit Bundle	ThermoScientific	901835
RC DC™ Protein Assay Kit	BioRad	5000121
NEBNext Poly(A) mRNA Magnetic Isolation Module	New England Biolabs	E7490S

NEBNext® Ultra™ II Directional RNA Library Prep Kit for Illumina®	New England Biolabs	E7760S
NextSeq 500/550 High Output 300 cycles kit	Illumina	20024908
**Software and algorithms**		
AGCC software	Affymetrix	N/A
Chromosome Analysis Suite (ChAS)	Affymetrix	software version 4.1
coadread_tcga_pan_can_atlas_2018	https://www.cell.com/pb-assets/consortium/pancanceratlas/pancani3/index.html">here	
Protein Pilot	SCIEX	Version 5.0
Peak View	SCIEX	Version 2.2
FASTQC	GitHub	Version 0.11.8
fastp	GitHub	Version 0.11
STAR	GitHub	Version 2.7.3a
RSEM	GitHub	Version 1.3.3
Tximport	GitHub	Version 1.16.1
UniProt ID mapping tool	https://www.uniprot.org/id-mapping	
STRING database	https://string-db.org	Version 12.0
Cytoscape	https://cytoscape.org	Version 3.10.2
GSEA	https://www.gsea-msigdb.org/gsea/index.jsp	Version 4.3.3
EMT signature	https://www.gsea-msigdb.org/gsea/msigdb/human/geneset/HALLMARK_EPITHELIAL_MESENCHYMAL_TRANSITION.html	Version 5.0
DepMap	https://depmap.org/portal/	
KMplot	https://kmplot.com/analysis/	
TNMplot	https://tnmplot.com/analysis/	
ISMARA	https://ismara.unibas.ch/mara/	
MitoCarta	https://www.broadinstitute.org/files/shared/metabolism/mitocarta/human.mitocarta3.0.html	Version 3.0
ImageJ	http://imageJ.nih.gov.ij	Version 1.53a
CellProfiler	Broad Institute https://cellprofiler.org	Version 4.2.8
**Deposited data**		
RNAseq	ENA	PRJEB57288
Mass spectrometry	ProteomeXchange Consortium, PRIDE	PXD038149
CytoscanHD	ENA	PRJEB75321

Table S2 Patient characteristics

ID sample	Tissue /PDOs	Gender	Ethnicity	PTL	CIN status	RAS RAF	MSS MSI	CNA analysis	Transcriptomic signature	Proteotranscriptomic integration
RTO2	+/+	F	Caucasian	rectum	CIN+	*KRAS* mut	MSS	yes	yes	yes
RTO7	+/+	M	Caucasian	rectum	CIN+	wt	MSS	yes	yes	yes
mCTO24S2	+/+	F	Caucasian	sigmoid	CIN-	*KRAS* mut	MSS	yes	yes	no
mCTO24S7	+/+	F	Caucasian	sigmoid	CIN-	*KRAS* mut	MSS	yes	no	no
mCTT24B	+/-	F	Caucasian	sigmoid	CIN+	*KRAS* mut	MSS	yes	no	no
mCTO38S5	+/+	M	Caucasian	rectum	CIN+	wt	MSS	yes	no	no
mCTT38S8	+/-	M	Caucasian	rectum	CIN+	wt	MSS	yes	no	no
mCTO43	+/+	M	Caucasian	sigmoid	CIN+	wt	MSS	yes	yes	yes
mCTO46	+/+	M	Caucasian	rectum	CIN+	*KRAS* mut	MSS	yes	no	no
mCTO47	+/+	M	Caucasian	sigmoid	CIN+	wt	MSS	yes	yes	no
mCTO50	-/+	F	Caucasian	cecum	CIN-	wt	MSI	yes	yes	yes
mCTO50B	+/+	F	Caucasian	cecum	CIN-	wt	MSI	yes	yes	yes
mCTO66S3	+/+	M	Caucasian	rectum	CIN+	*KRAS* mut	MSS	yes	yes	yes
mCTO66S7	+/+	M	Caucasian	rectum	CIN+	*KRAS* mut	MSS	yes	no	no

Figure S1

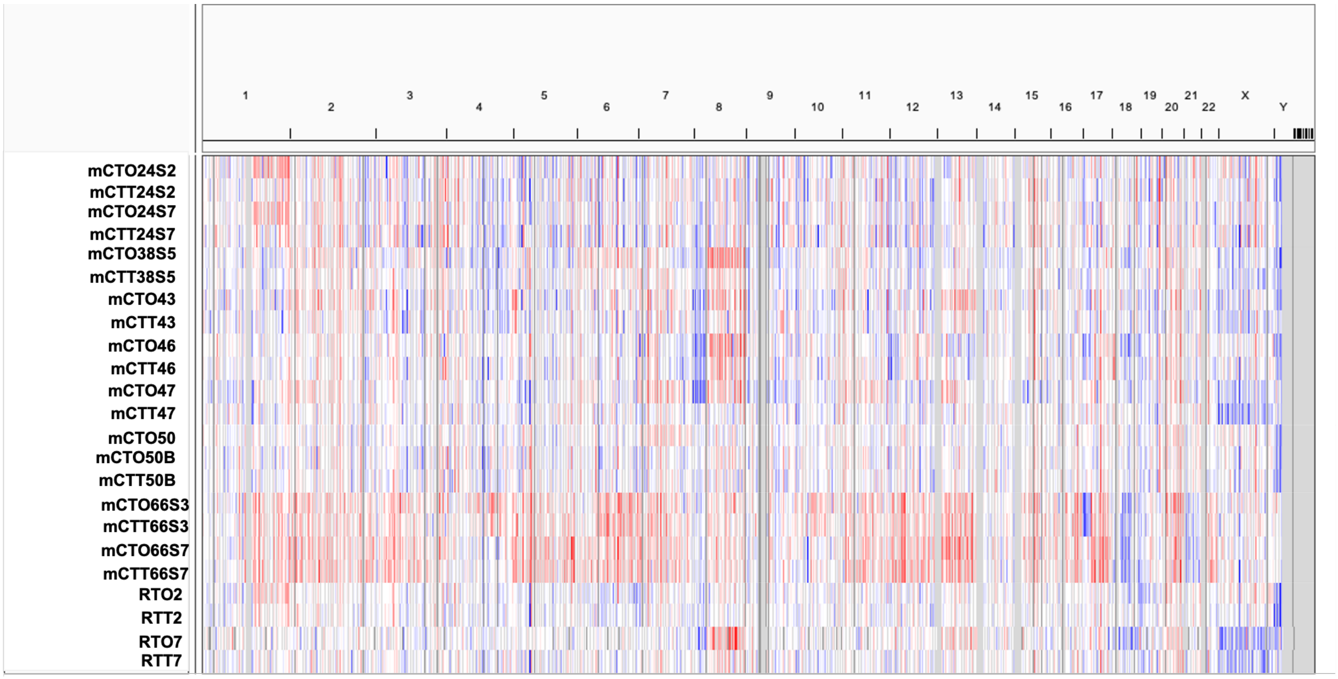

1
2
3
4
5
6
7
8
9
10
11
12
13
14
15
16
17
18
19
20
21
22
X
Y
mCTO24S2
mCTT24S2
mCTO24S7
mCTT24S7
mCTO38S5
mCTT38S5
mCTO43
mCTT43
mCTO46
mCTT46
mCTO47
mCTT47
mCTO50
mCTO50B
mCTT50B
mCTO66S3
mCTT66S3
mCTO66S7
mCTT66S7
RTO2
RTT2
RTO7
RTT7

Figure S2

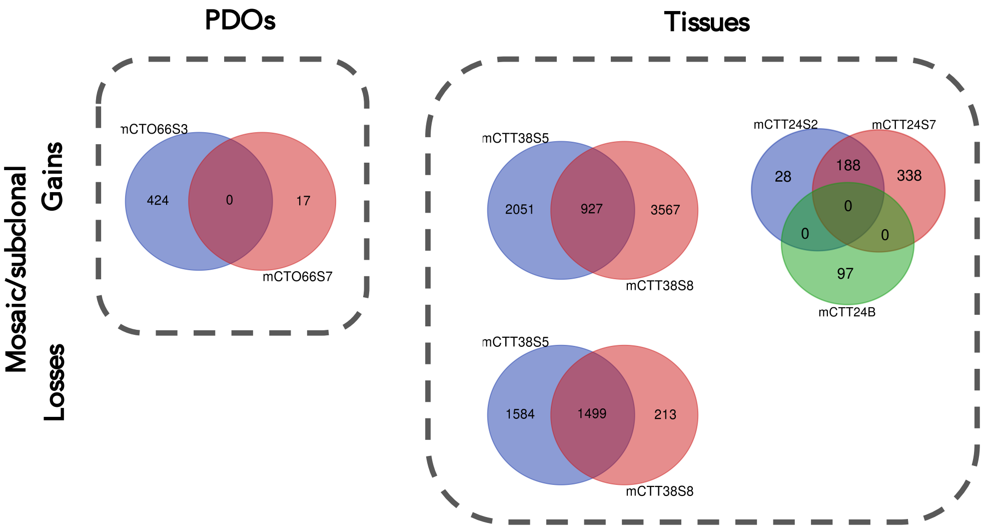
PDOs
Tissues
Mosaic/subclonal
Gains
Losses
mCTO66S3
424
0
17
mCTO66S7
mCTT38S5
2051
927
3567
mCTT38S8
mCTT24S2
mCTT24S7
28
188
338
0
0
0
97
mCTT24B
mCTT38S5
1584
1499
213
mCTT38S8

Figure S3

a

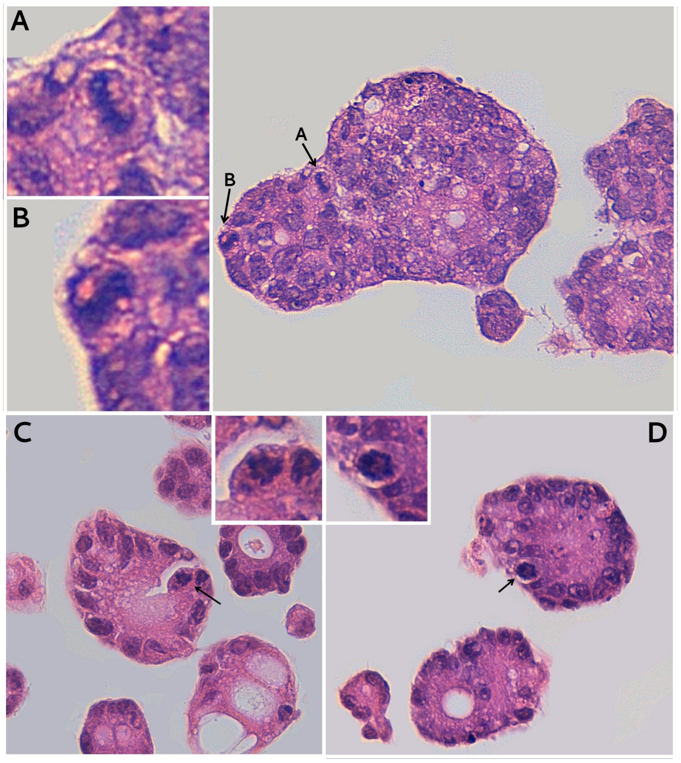

A
B
C
D

b

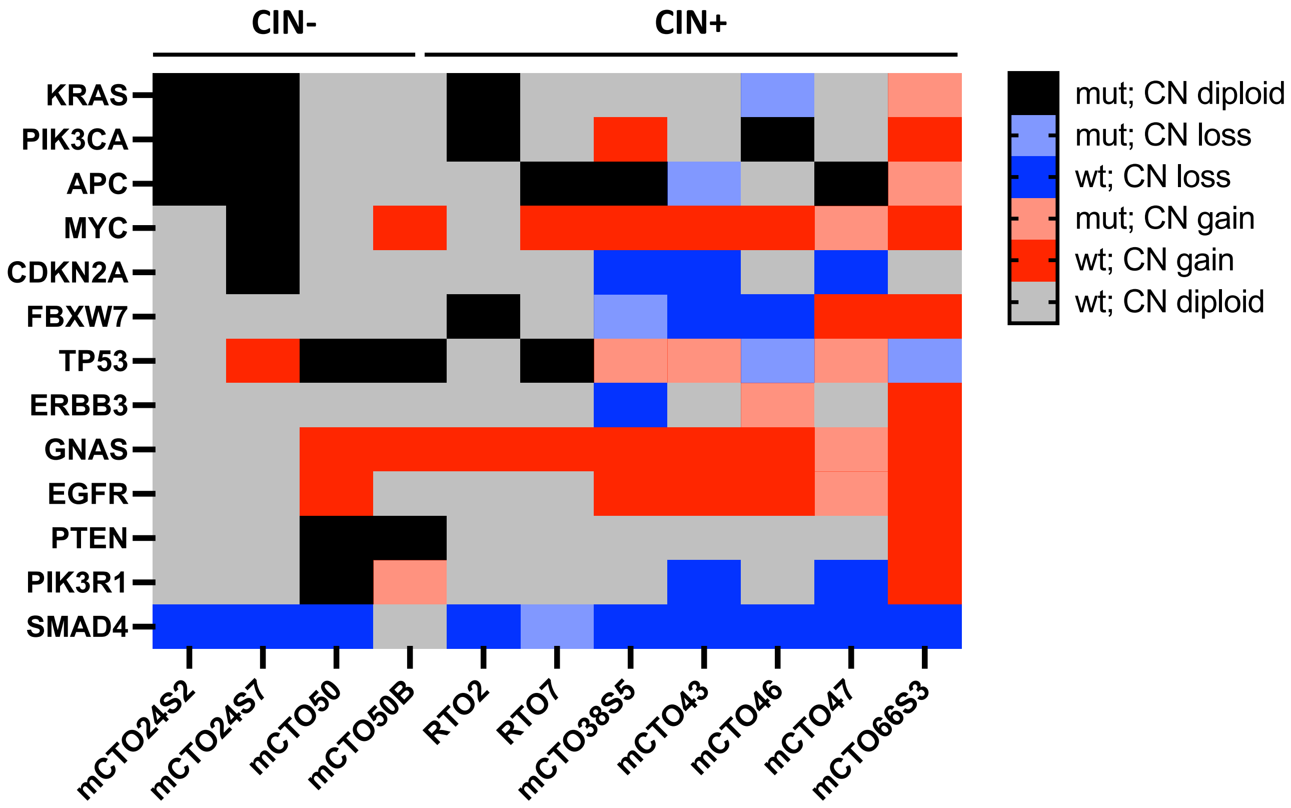

CIN-
CIN+
KRAS
PIK3CA
APC
MYC
CDKN2A
FBXW7
TP53
ERBB3
GNAS
EGFR
PTEN
PIK3R1
SMAD4
mCTO24S2
mCTO24S7
mCTO50
mCTO50B
RTO2
RTO7
mCTO38S5
mCTO43
mCTO46
mCTO47
mCTO66S3
mut; CN diploid
mut; CN loss
wt; CN loss
mut; CN gain
wt; CN gain
wt; CN diploid

Figure S4

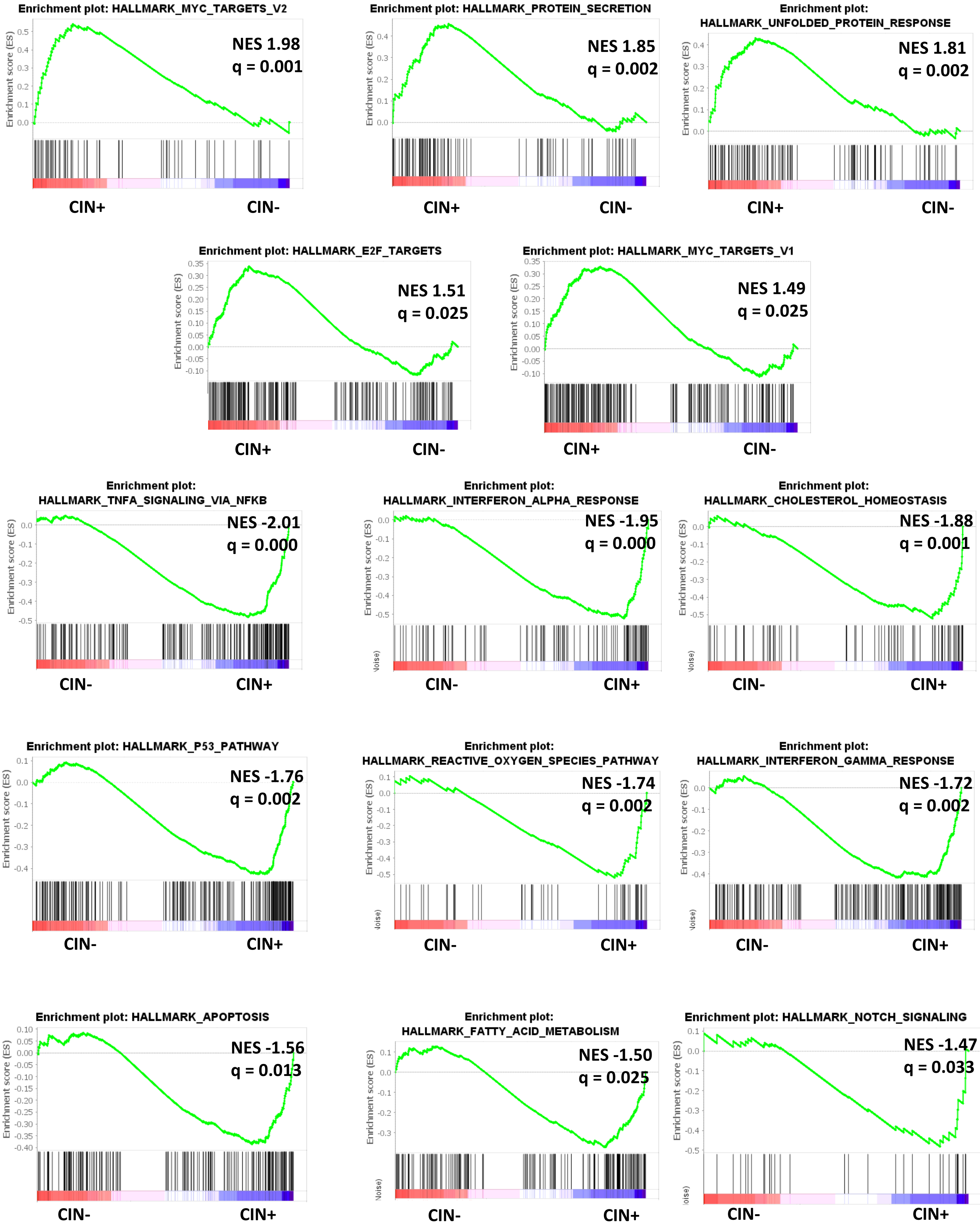
Enrichment plot: HALLMARK_MYC_TARGETS_V2
NES 1.98
q = 0.001
CIN+
CIN-
Enrichment plot: HALLMARK_PROTEIN_SECRETION
NES 1.85
q = 0.002
CIN+
CIN-
Enrichment plot: HALLMARK_UNFOLDED_PROTEIN_RESPONSE
NES 1.81
q = 0.002
CIN+
CIN-
Enrichment plot: HALLMARK_E2F_TARGETS
NES 1.51
q = 0.025
CIN+
CIN-
Enrichment plot: HALLMARK_MYC_TARGETS_V1
NES 1.49
q = 0.025
CIN+
CIN-
Enrichment plot: HALLMARK_TNFA_SIGNALING_VIA_NFKB
NES -2.01
q = 0.000
CIN-
CIN+
Enrichment plot: HALLMARK_INTERFERON_ALPHA_RESPONSE
NES -1.95
q = 0.000
CIN-
CIN+
Enrichment plot: HALLMARK_CHOLESTEROL_HOMEOSTASIS
NES -1.88
q = 0.001
CIN-
CIN+
Enrichment plot: HALLMARK_P53_PATHWAY
NES -1.76
q = 0.002
CIN-
CIN+
Enrichment plot: HALLMARK_REACTIVE_OXYGEN_SPECIES_PATHWAY
NES -1.74
q = 0.002
CIN-
CIN+
Enrichment plot: HALLMARK_INTERFERON_GAMMA_RESPONSE
NES -1.72
q = 0.002
CIN-
CIN+
Enrichment plot: HALLMARK_APOPTOSIS
NES -1.56
q = 0.013
CIN-
CIN+
Enrichment plot: HALLMARK_FATTY_ACID_METABOLISM
NES -1.50
q = 0.025
CIN-
CIN+
Enrichment plot: HALLMARK_NOTCH_SIGNALING
NES -1.47
q = 0.033
CIN-
CIN+
Enrichment score (ES)

Figure S5

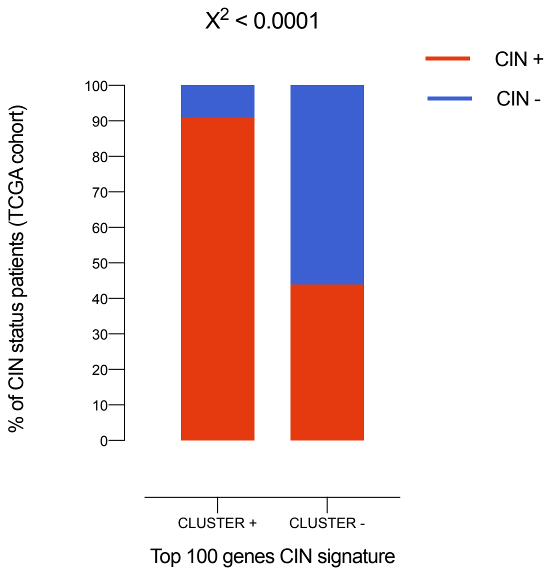
X2 < 0.0001
CIN +
CIN -
% of CIN status patients (TCGA cohort)
100
90
80
70
60
50
40
30
20
10
0
CLUSTER +
CLUSTER -
Top 100 genes CIN signature

Figure S6

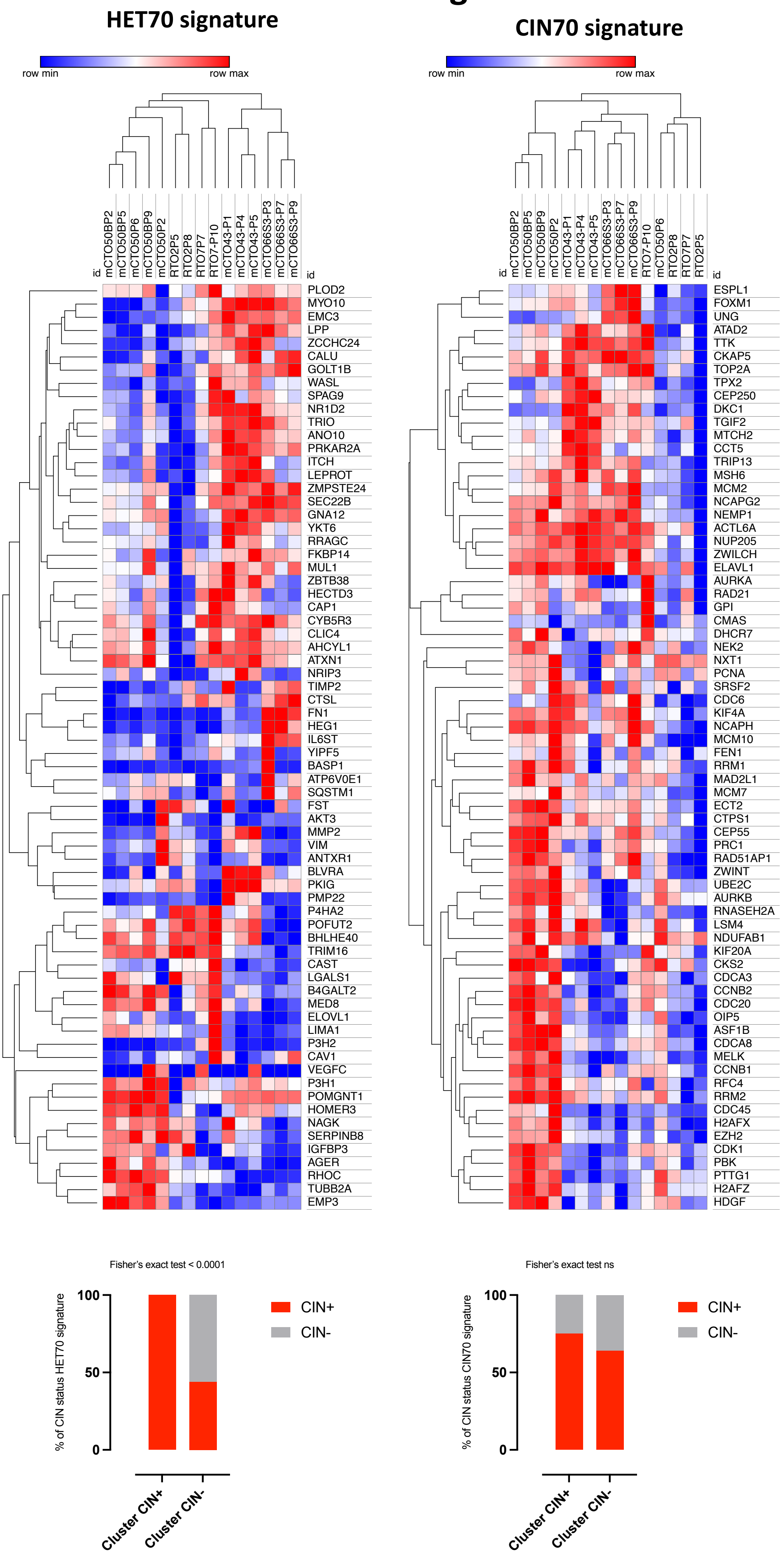
HET70 signature
row min
row max
id
mCTO50BP2
mCTO50BP5
mCTO50P6
mCTO50BP9
mCTO50P2
RTO2P5
RTO2P8
RTO7P7
RTO7-P10
mCTO43-P1
mCTO43-P4
mCTO43-P5
mCTO66S3-P3
mCTO66S3-P7
mCTO66S3-P9
id
PLOD2
MYO10
EMC3
LPP
ZCCHC24
CALU
GOLT1B
WASL
SPAG9
NR1D2
TRIO
ANO10
PRKAR2A
ITCH
LEPROT
ZMPSTE24
SEC22B
GNA12
YKT6
RRAGC
FKBP14
MUL1
ZBTB38
HECTD3
CAP1
CYB5R3
CLIC4
AHCYL1
ATXN1
NRIP3
TIMP2
CTSL
FN1
HEG1
IL6ST
YIPF5
BASP1
ATP6V0E1
SQSTM1
FST
AKT3
MMP2
VIM
ANTXR1
BLVRA
PKIG
PMP22
P4HA2
POFUT2
BHLHE40
TRIM16
CAST
LGALS1
B4GALT2
MED8
ELOVL1
LIMA1
P3H2
CAV1
VEGFC
P3H1
POMGNT1
HOMER3
NAGK
SERPINB8
IGFBP3
AGER
RHOC
TUBB2A
EMP3
CIN70 signature
row min
row max
id
mCTO50BP2
mCTO50BP5
mCTO50BP9
mCTO50P2
mCTO43-P1
mCTO43-P4
mCTO43-P5
mCTO66S3-P3
mCTO66S3-P7
mCTO66S3-P9
RTO7-P10
mCTO50P6
RTO2P8
RTO7P7
RTO2P5
id
ESPL1
FOXM1
UNG
ATAD2
TTK
CKAP5
TOP2A
TPX2
CEP250
DKC1
TGIF2
MTCH2
CCT5
TRIP13
MSH6
MCM2
NCAPG2
NEMP1
ACTL6A
NUP205
ZWILCH
ELAVL1
AURKA
RAD21
GPI
CMAS
DHCR7
NEK2
NXT1
PCNA
SRSF2
CDC6
KIF4A
NCAPH
MCM10
FEN1
RRM1
MAD2L1
MCM7
ECT2
CTPS1
CEP55
PRC1
RAD51AP1
ZWINT
UBE2C
AURKB
RNASEH2A
LSM4
NDUFAB1
KIF20A
CKS2
CDCA3
CCNB2
CDC20
OIP5
ASF1B
CDCA8
MELK
CCNB1
RFC4
RRM2
CDC45
H2AFX
EZH2
CDK1
PBK
PTTG1
H2AFZ
HDGF
Fisher's exact test < 0.0001
% of CIN status HET70 signature
100
50
0
CIN+
CIN-
Cluster CIN+
Cluster CIN-
Fisher's exact test ns
% of CIN status CIN70 signature
100
50
0
CIN+
CIN-
Cluster CIN+
Cluster CIN-

Figure S7

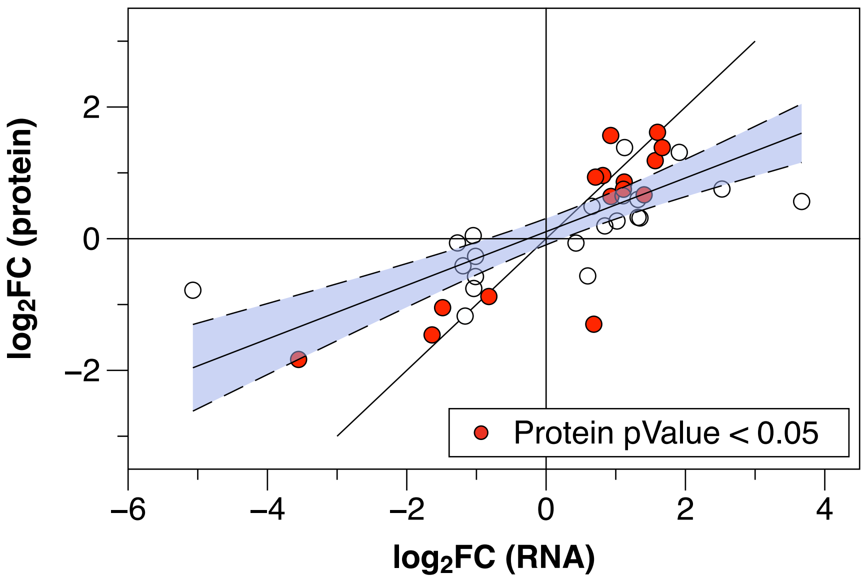

Figure S8

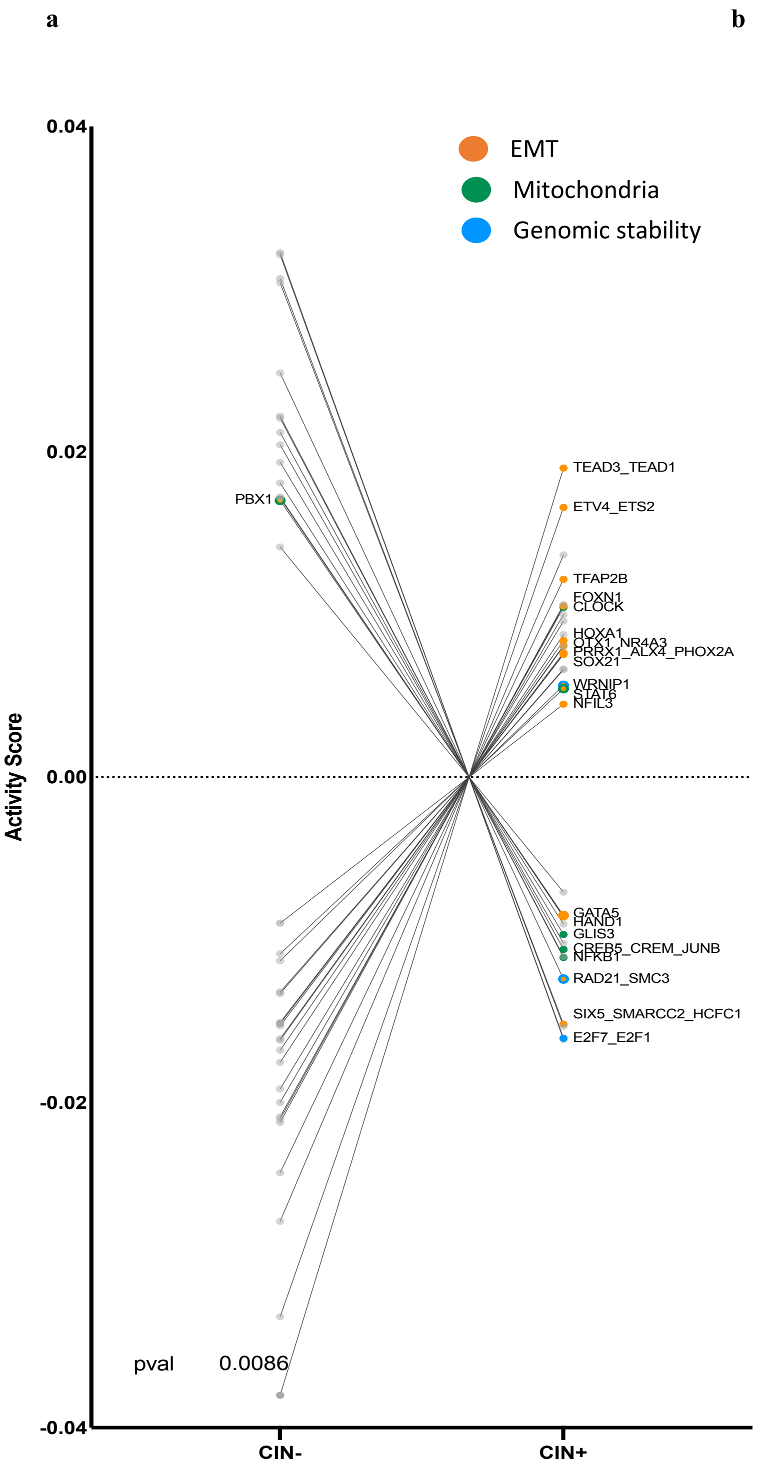

a
EMT
Mitochondria
Genomic stability
Activity Score
0.04
0.02
0.00
-0.02
-0.04
PBX1
TEAD3_TEAD1
ETV4_ETS2
TFAP2B
FOXN1
CLOCK
HOXA1
OTX1_NR4A3
PRRX1_ALX4_PHOX2A
SOX21
WRNIP1
STAT6
NFIL3
GATA5
HAND1
GLIS3
CREB5_CREM_JUNB
NFKB1
RAD21_SMC3
SIX5_SMARCC2_HCFC1
E2F7_E2F1
pval 0.0086
CIN-
CIN+

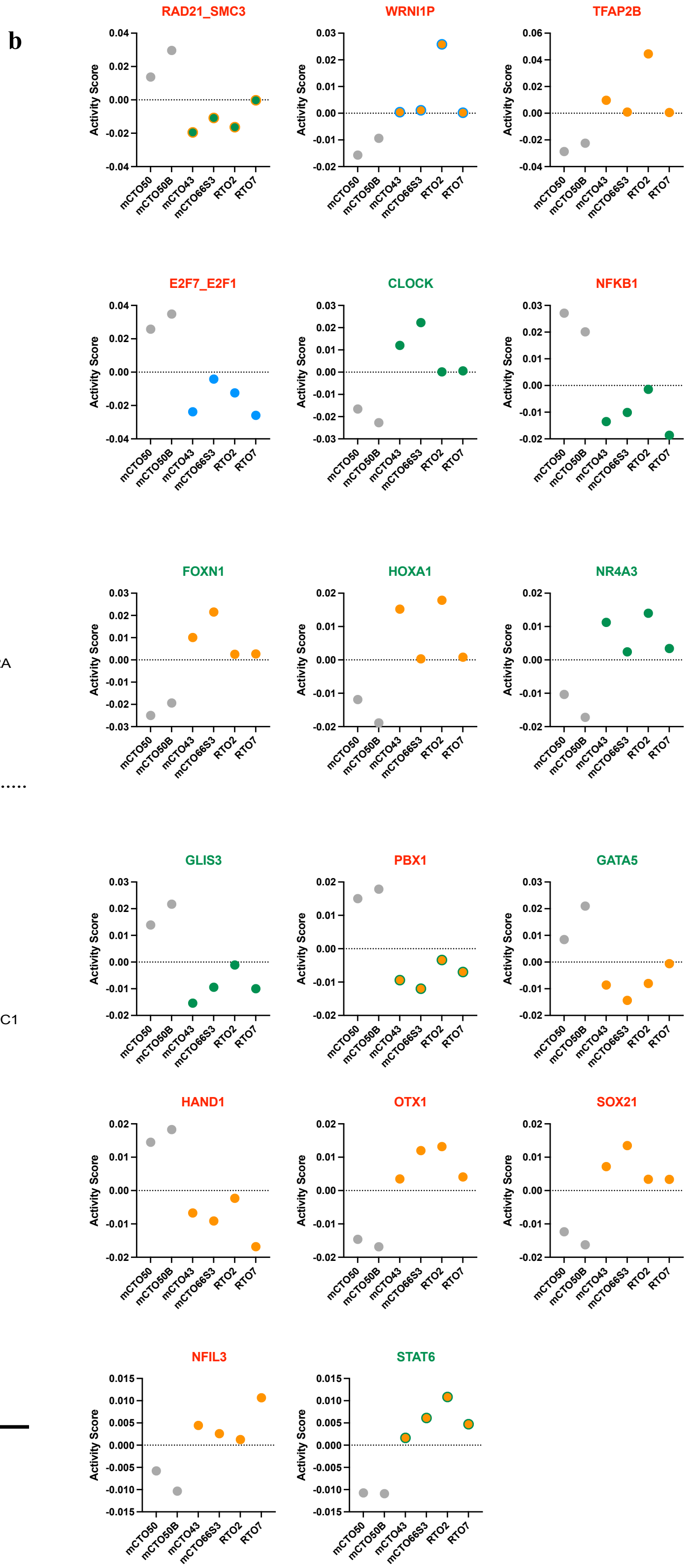

b
RAD21_SMC3
WRNI1P
TFAP2B
E2F7_E2F1
CLOCK
NFKB1
FOXN1
HOXA1
NR4A3
GLIS3
PBX1
GATA5
HAND1
OTX1
SOX21
NFIL3
STAT6
Activity Score
mCTO50
mCTO50B
mCTO43
mCTO66S3
RTO2
RTO7

Figure S9

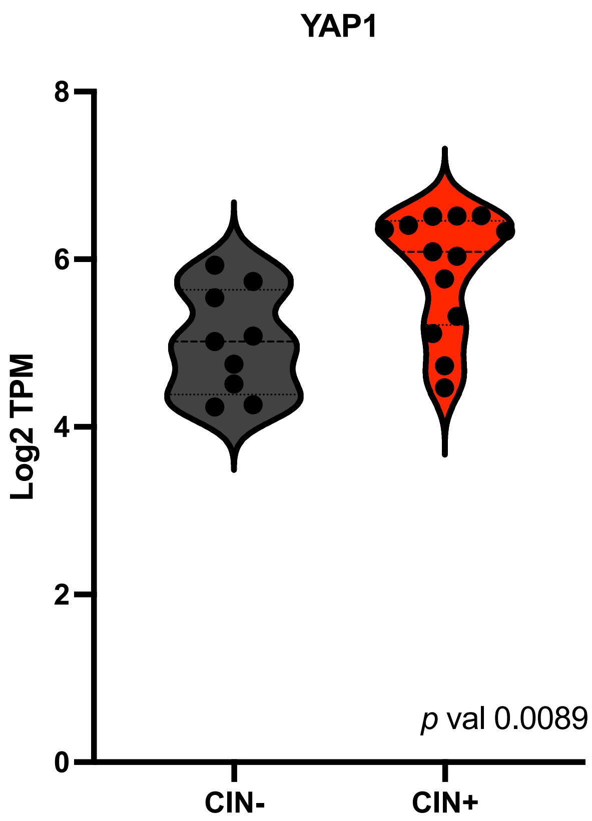

Figure S10

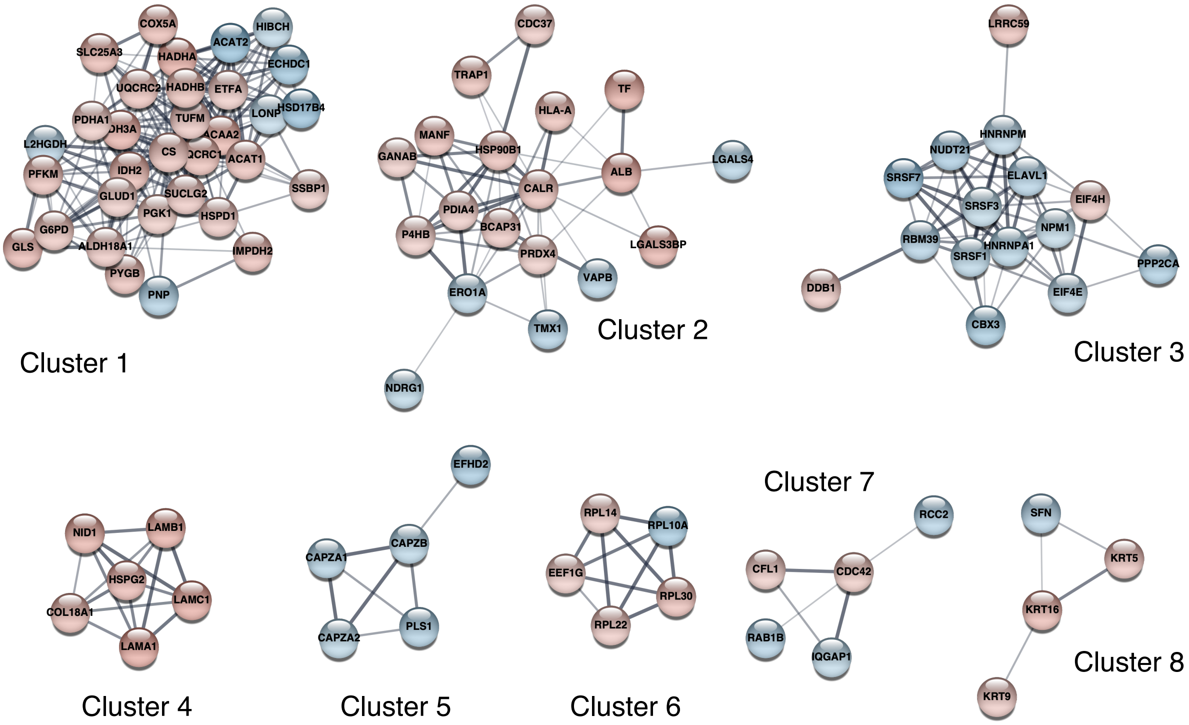

COX5A
HIBCH
SLC25A3
ACAT2
HADHA
ECHDC1
UQCRC2
HADHB
ETFA
HSD17B4
PDHA1
TUFM
L2HGDH
CS
ACAT1
PFKM
IDH2
SSBP1
GLUD1
SUCLG2
PGK1
HSPD1
G6PD
GLS
ALDH18A1
IMPDH2
PYGB
PNP
Cluster 1
CDC37
TRAP1
TF
HLA-A
MANF
HSP90B1
GANAB
ALB
LGALS4
CALR
PDIA4
BCAP31
P4HB
LGALS3BP
PRDX4
VAPB
ERO1A
TMX1
Cluster 2
NDRG1
LRRC59
HNRNPM
NUDT21
SRSF7
ELAVL1
EIF4H
SRSF3
NPM1
RBM39
HNRNPA1
SRSF1
PPP2CA
DDB1
EIF4E
CBX3
Cluster 3
EFHD2
NID1
LAMB1
HSPG2
COL18A1
LAMC1
LAMA1
Cluster 4
CAPZA1
CAPZB
CAPZA2
PLS1
Cluster 5
RPL14
RPL10A
EEF1G
RPL30
RPL22
Cluster 6
Cluster 7
RCC2
CFL1
CDC42
RAB1B
IQGAP1
SFN
KRT5
KRT16
KRT9
Cluster 8

a

Figure S11

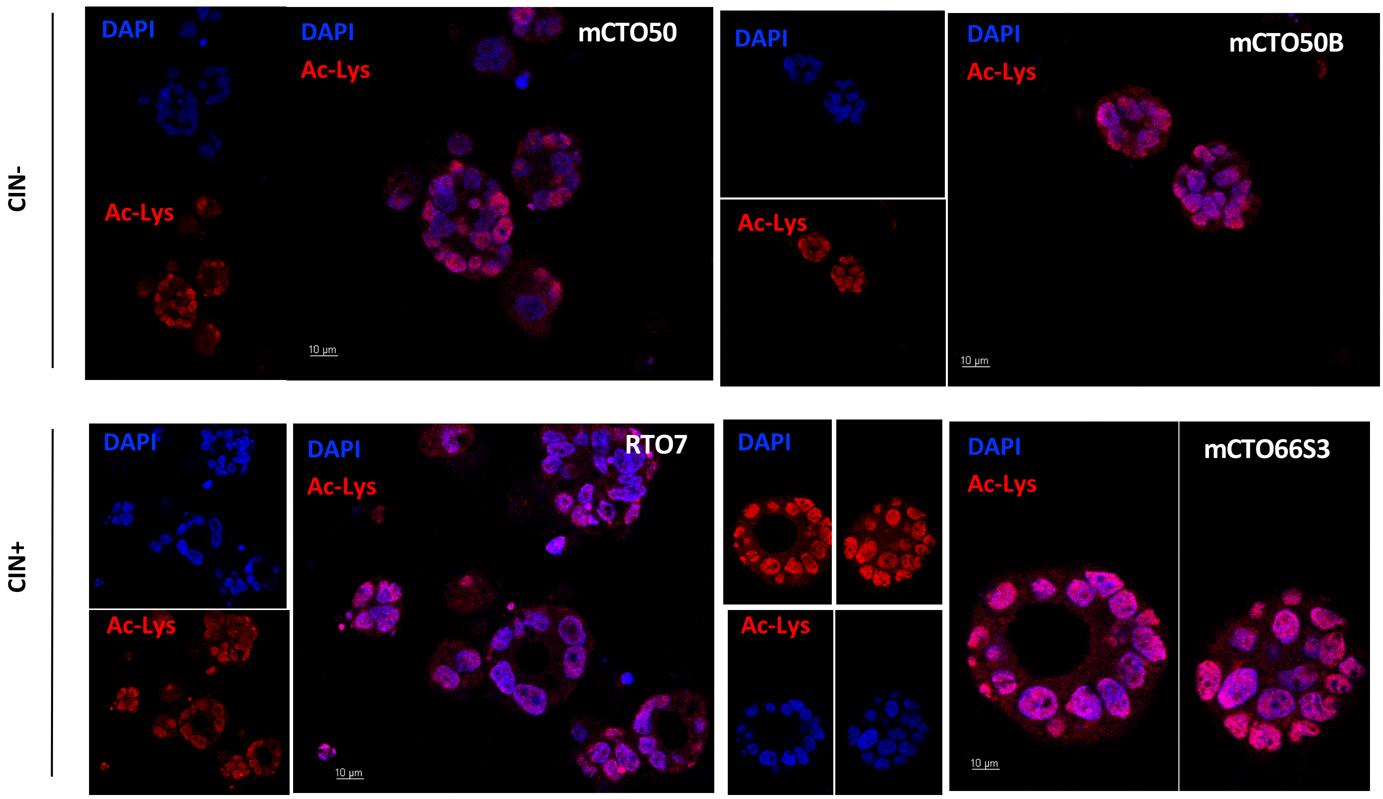

b

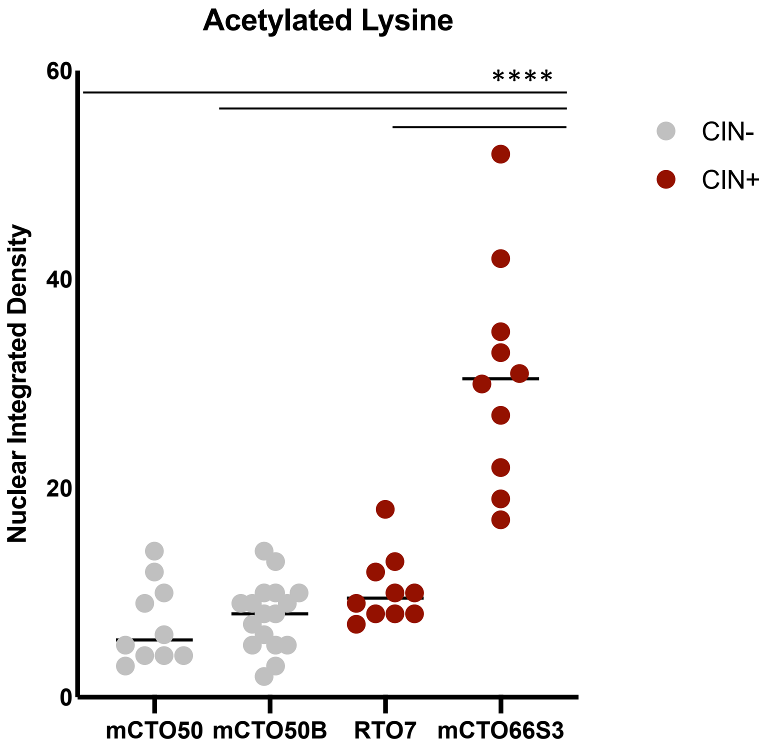

Figure S12

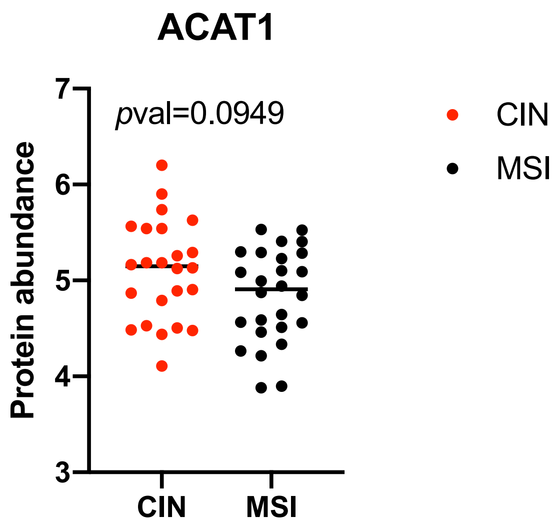

Figure S13

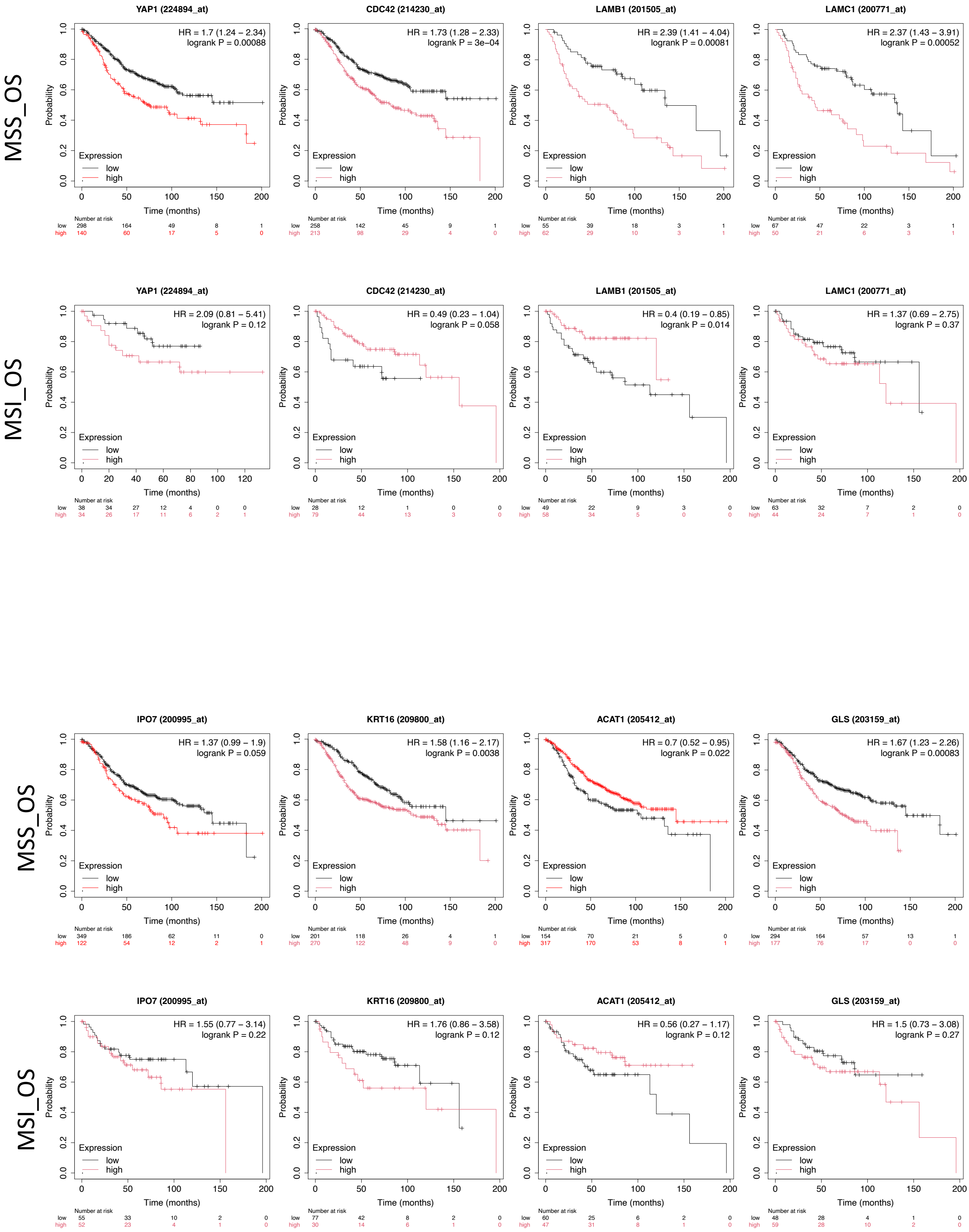
MSS_OS
YAP1 (224894_at)
HR = 1.7 (1.24 – 2.34)
logrank P = 0.00088
CDC42 (214230_at)
HR = 1.73 (1.28 – 2.33)
logrank P = 3e−04
LAMB1 (201505_at)
HR = 2.39 (1.41 – 4.04)
logrank P = 0.00081
LAMC1 (200771_at)
HR = 2.37 (1.43 – 3.91)
logrank P = 0.00052
MSI_OS
YAP1 (224894_at)
HR = 2.09 (0.81 – 5.41)
logrank P = 0.12
CDC42 (214230_at)
HR = 0.49 (0.23 – 1.04)
logrank P = 0.058
LAMB1 (201505_at)
HR = 0.4 (0.19 – 0.85)
logrank P = 0.014
LAMC1 (200771_at)
HR = 1.37 (0.69 – 2.75)
logrank P = 0.37
MSS_OS
IPO7 (200995_at)
HR = 1.37 (0.99 – 1.9)
logrank P = 0.059
KRT16 (209800_at)
HR = 1.58 (1.16 – 2.17)
logrank P = 0.0038
ACAT1 (205412_at)
HR = 0.7 (0.52 – 0.95)
logrank P = 0.022
GLS (203159_at)
HR = 1.67 (1.23 – 2.26)
logrank P = 0.00083
MSI_OS
IPO7 (200995_at)
HR = 1.55 (0.77 – 3.14)
logrank P = 0.22
KRT16 (209800_at)
HR = 1.76 (0.86 – 3.58)
logrank P = 0.12
ACAT1 (205412_at)
HR = 0.56 (0.27 – 1.17)
logrank P = 0.12
GLS (203159_at)
HR = 1.5 (0.73 – 3.08)
logrank P = 0.27
Probability
Time (months)
Expression
low
high
Number at risk

Figure S14

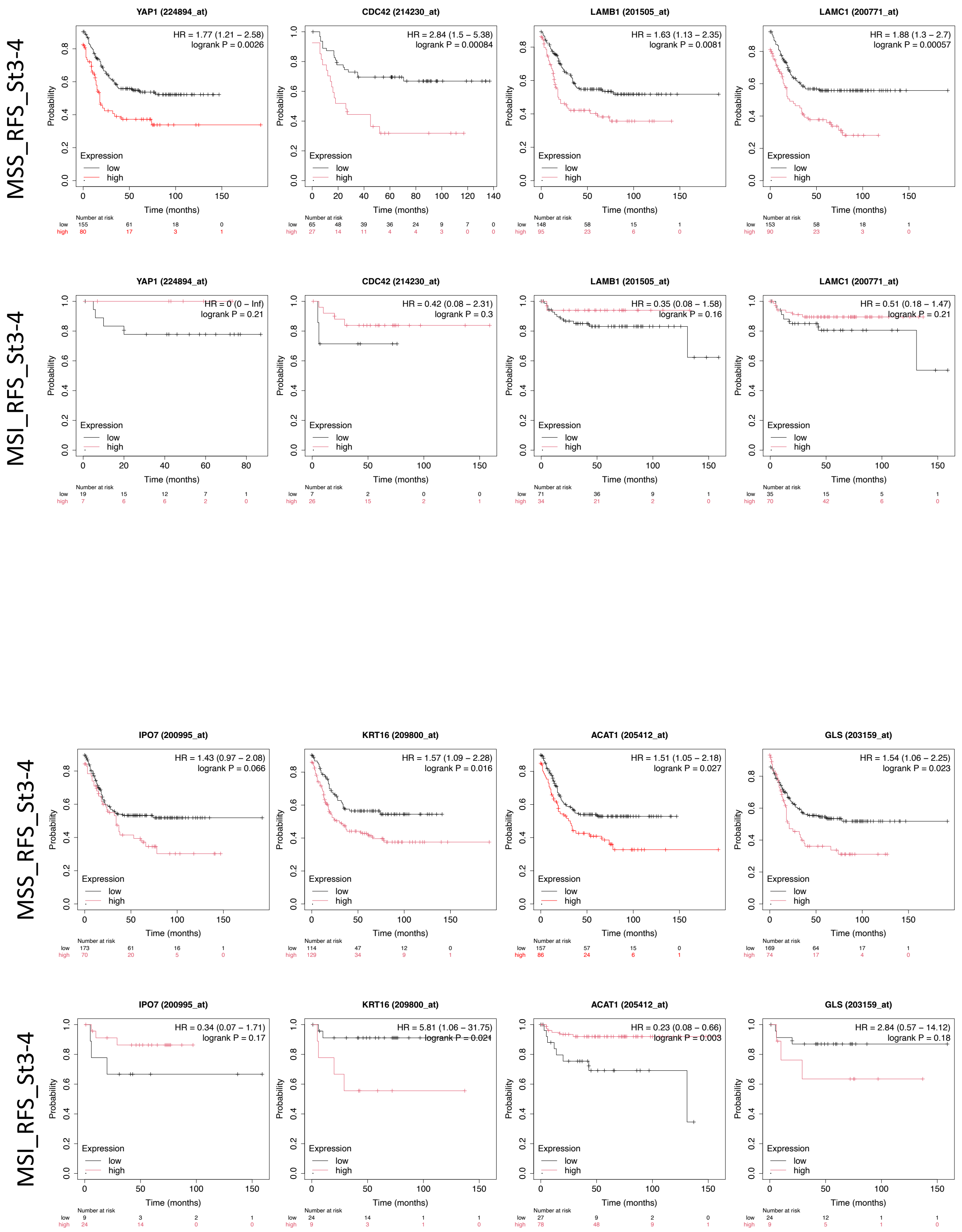
MSS_RFS_St3-4
YAP1 (224894_at)
HR = 1.77 (1.21 – 2.58)
logrank P = 0.0026
CDC42 (214230_at)
HR = 2.84 (1.5 – 5.38)
logrank P = 0.00084
LAMB1 (201505_at)
HR = 1.63 (1.13 – 2.35)
logrank P = 0.0081
LAMC1 (200771_at)
HR = 1.88 (1.3 – 2.7)
logrank P = 0.00057
MSI_RFS_St3-4
YAP1 (224894_at)
HR = 0 (0 – Inf)
logrank P = 0.21
CDC42 (214230_at)
HR = 0.42 (0.08 – 2.31)
logrank P = 0.3
LAMB1 (201505_at)
HR = 0.35 (0.08 – 1.58)
logrank P = 0.16
LAMC1 (200771_at)
HR = 0.51 (0.18 – 1.47)
logrank P = 0.21
MSS_RFS_St3-4
IPO7 (200995_at)
HR = 1.43 (0.97 – 2.08)
logrank P = 0.066
KRT16 (209800_at)
HR = 1.57 (1.09 – 2.28)
logrank P = 0.016
ACAT1 (205412_at)
HR = 1.51 (1.05 – 2.18)
logrank P = 0.027
GLS (203159_at)
HR = 1.54 (1.06 – 2.25)
logrank P = 0.023
MSI_RFS_St3-4
IPO7 (200995_at)
HR = 0.34 (0.07 – 1.71)
logrank P = 0.17
KRT16 (209800_at)
HR = 5.81 (1.06 – 31.75)
logrank P = 0.021
ACAT1 (205412_at)
HR = 0.23 (0.08 – 0.66)
logrank P = 0.003
GLS (203159_at)
HR = 2.84 (0.57 – 14.12)
logrank P = 0.18
Probability
Time (months)
Expression
low
high
Number at risk

Figure S15

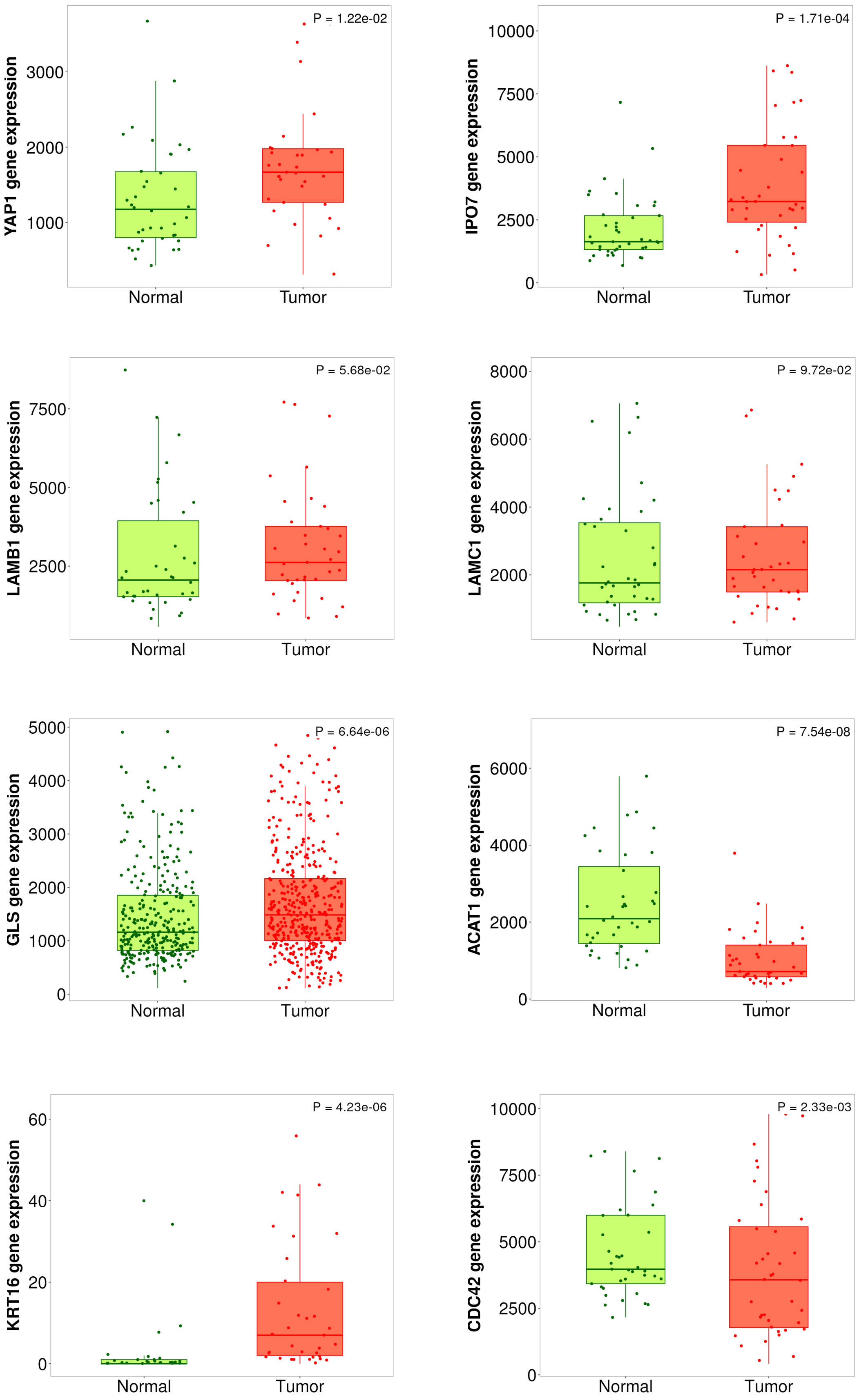

P = 1.22e-02
YAP1 gene expression
3000
2000
1000
Normal
Tumor
P = 1.71e-04
IPO7 gene expression
10000
7500
5000
2500
0
Normal
Tumor
P = 5.68e-02
LAMB1 gene expression
7500
5000
2500
Normal
Tumor
P = 9.72e-02
LAMC1 gene expression
8000
6000
4000
2000
Normal
Tumor
P = 6.64e-06
GLS gene expression
5000
4000
3000
2000
1000
0
Normal
Tumor
P = 7.54e-08
ACAT1 gene expression
6000
4000
2000
0
Normal
Tumor
P = 4.23e-06
KRT16 gene expression
60
40
20
0
Normal
Tumor
P = 2.33e-03
CDC42 gene expression
10000
7500
5000
2500
0
Normal
Tumor
